# Evaluation of Ecotoxicology Assessment Methods of Nanomaterials and Their Effects

**DOI:** 10.3390/nano10040610

**Published:** 2020-03-26

**Authors:** Bianca-Vanesa Boros, Vasile Ostafe

**Affiliations:** Advanced Environmental Research Laboratories, Department of Biology—Chemistry, West University of Timisoara, Oituz 4, 300086 Timisoara, Romania; bianca.boros91@e-uvt.ro

**Keywords:** nanomaterials, organisms suitable for simple and rapid ecotoxicity testing SSRET, ecotoxicological test batteries, ecotoxicology

## Abstract

This paper describes the ecotoxicological effects of nanomaterials (NMs) as well as their testing methods. Standard ecotoxicity testing methods are applicable to nanomaterials as well but require some adaptation. We have taken into account methods that meet several conditions. They must be properly researched by a minimum of ten scientific articles where adaptation of the method to the NMs is also presented; use organisms suitable for simple and rapid ecotoxicity testing (SSRET); have a test period shorter than 30 days; require no special equipment; have low costs and have the possibility of optimization for high-throughput screening. From the standard assays described in guidelines developed by organizations such as Organization for Economic Cooperation and Development and United States Environmental Protection Agency, which meet the required conditions, we selected as methods adaptable for NMs, some methods based on algae, duckweed, amphipods, daphnids, chironomids, terrestrial plants, nematodes and earthworms. By analyzing the effects of NMs on a wide range of organisms, it has been observed that these effects can be of several categories, such as behavioral, morphological, cellular, molecular or genetic effects. By comparing the EC_50_ values of some NMs it has been observed that such values are available mainly for aquatic ecotoxicity, with the most sensitive test being the algae assay. The most toxic NMs overall were the silver NMs.

## 1. Introduction

**Figure 1:**
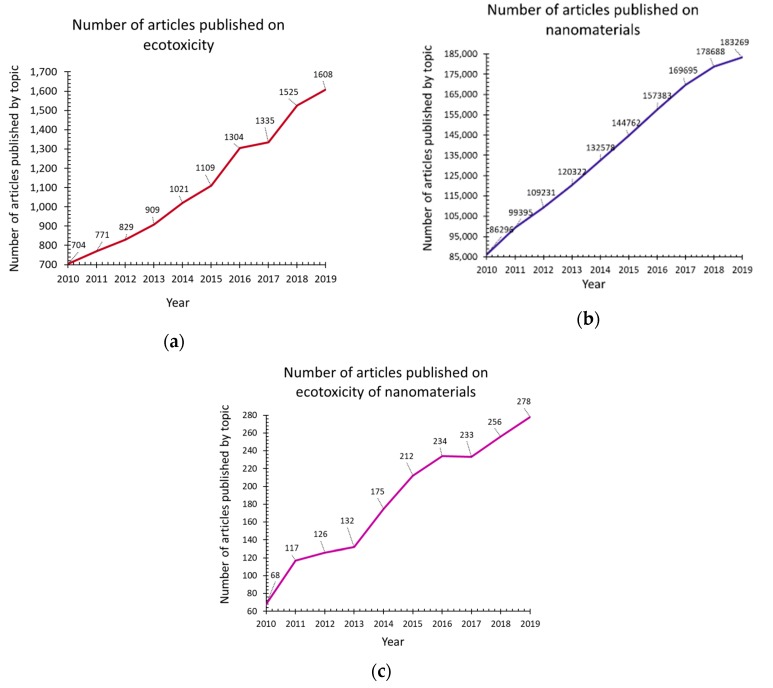
Bibliometric analysis of research in 2010–2019 from Web of Science Core Collection (Web of Science, Clarivate Analytics) on (**a**) ecotoxicity (search strategy: topic search (TS)=(Ecotox*) (containing “ecotox” in the topic of the articles)); (**b**) nanomaterials (search strategy: TS=(Nano*)) and (**c**) ecotoxicity of nanomaterials (search strategy: TS=(Ecotox* AND Nano*)).

Both the study of nanomaterials and that of ecotoxicology are constantly increasing, as evidenced by the increasing number of scientific articles from Web of Science Core Collection (Web of Science, Clarivate Analytics) during 2010–2019 (Figure 1a,b). Due to the wide range of applications of nanomaterials, as well as the increased importance of protecting the environment and analyzing the ecotoxicity of potential pollutants, investigating the ecotoxicological effects of nanomaterials is essential. This is also underlined by the increasing number of scientific articles on the ecotoxicity of nanomaterials from Web of Science Core Collection (Web of Science, Clarivate Analytics) in 2010–2019 (Figure 1c). With the emergence of the need to assess the ecotoxicity of nanomaterials, the need arises to adapt standard ecotoxicity tests for testing nanomaterials.

In the present work, a series of bibliometric investigations were carried out to observe and analyze the state of the research on the ecotoxicity, nanomaterials and the ecotoxicity of the nanomaterials (Table 1.). These studies have highlighted the importance of research on the ecotoxicity of nanomaterials, the adaptation of the standard methods for testing the ecotoxicity of nanomaterials, and also highlighted the need to study the ecotoxicity in case of less studied nanomaterials. All bibliometric data were obtained from Web of Science Core Collection (Web of Science, Clarivate Analytics) in January 2020.

In this paper we address both the adaptation of the standard ecotoxicity testing methods for nanomaterials, as well as the mechanisms of the ecotoxicological effects of these nanomaterials described as their biological and biochemical effects.

### 1.1. Ecotoxicity

The term “ecotoxicology” was first used by Ernst Haeckel in 1866 in the sense of the “economy of nature” and the “science of all interactions between organisms and their environment”. In the following years the science of ecotoxicology was developed as the study, using the tools of toxicology, of the effects of radiation and chemical pollutants from the biosphere on organisms from the environment [1,2]. In 1969, René Truhaut defined the term “ecotoxicology” as “the branch of toxicology concerned with the study of toxic effects, caused by natural or synthetic pollutants, to the constituents of ecosystems, animal, vegetable and microbial, in an integral context” [2].

The ecotoxicological research is under rapid development due to the pollution of the environment caused by the rapid industrial development and it is speeded up by severe industrial accidents. Since ecotoxicology became an important part in ecological and environmental risk assessment, ecotoxicity assessment policies were developed [2].

The environment hazard assessment framework uses ecotoxicity tests which are tools used to answer questions about the dangers of chemical substances that may be released into the environment [3].

**Figure 2:**
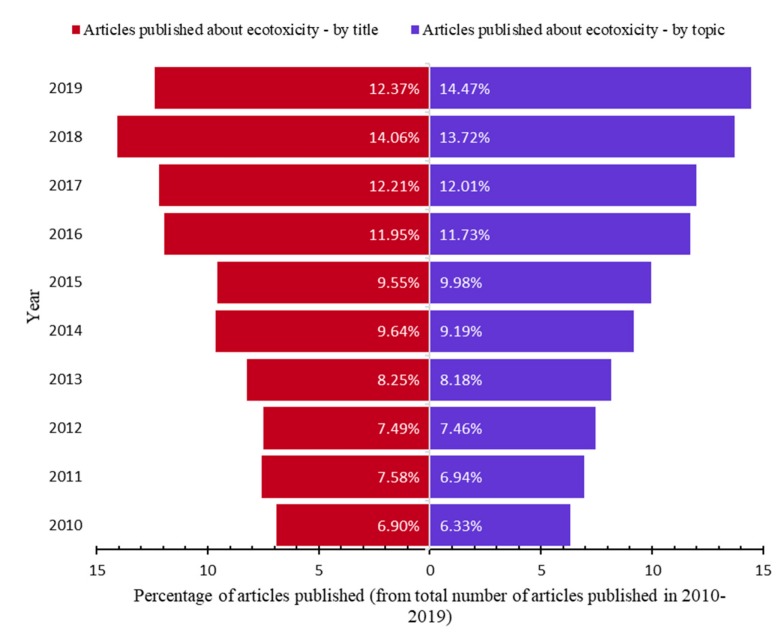
Bibliometric analysis of research on ecotoxicity in 2010–2019 from Web of Science Core Collection (Web of Science, Clarivate Analytics), the search strategy used to retrieve the data being: title search (TI)=(Ecotox*) (containing “ecotox” in the title of the articles) and TS=(Ecotox*) (containing “ecotox” in the topic of the articles).

The percentage of scientific articles by year in the Web of Science Core Collection (Web of Science, Clarivate Analytics) from the total of articles in 2010–2019 is increasing (Figure 2). This highlights the increasing importance of ecotoxicological testing in all areas and the necessity of adapting these assays to the new types of materials.

**Figure 3:**
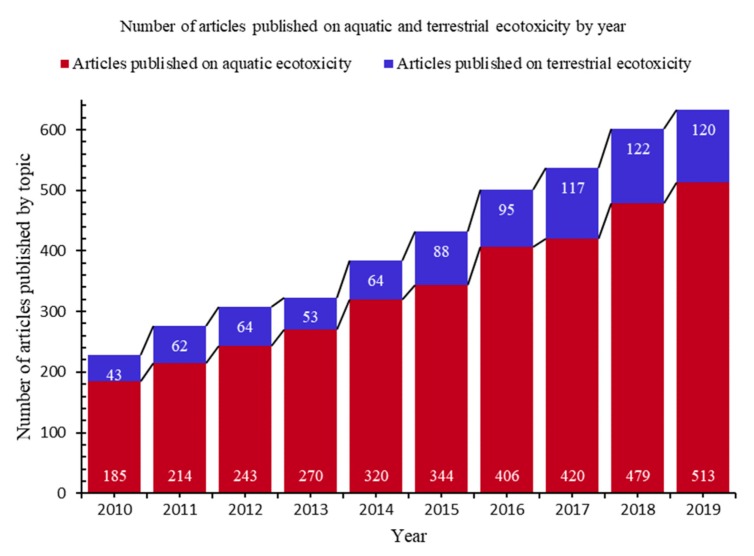
Bibliometric analysis of research on aquatic and terrestrial ecotoxicity in 2010–2019 from Web of Science Core Collection (Web of Science, Clarivate Analytics), the search strategy used to retrieve the data being: TS=(aquat* AND ecotox*) (containing both “aquat” and “ecotox” in the topic of the articles) and TS=(terrestr* AND ecotox*).

The ecotoxicological assessment developed as aquatic and terrestrial, the terrestrial assessment lagging the aquatic one [2]. Although the number of scientific articles published both in the subject of aquatic ecotoxicity and in terrestrial ecotoxicity is increasing, the ratio between the two types remains relatively constant even during the period 2010–2019, the aquatic ecotoxicity being more researched than the terrestrial one (Figure 3). This highlights the necessity to further develop the study on terrestrial ecotoxicity to reach the level of the aquatic one, or at least to get close to its level.

The ultimate concern of ecotoxicology is the establishment of the consequences of the effects at population level and on whole ecosystems, but in practice much work is done at individual level [4].

Standardized tests under the forms of guidelines were formulated by different organizations, such as Organization for Economic Cooperation and Development (OECD), United States Environmental Protection Agency (EPA), International Organization for Standardization (ISO), Government of Canada (GC) or American Society for Testing and Materials (ASTM). The standardized assay can be conducted on:

- aquatic organisms
  ○ algae
  ○ plants
  ○ invertebrates
  ○ vertebrates
- terrestrial organisms
  ○ algae
  ○ plants
  ○ invertebrates
  ○ vertebrates

These assays use a wide range of organisms depending on the type of test needed. A list of these organisms is provided in Table 2 and Table 3.

In Europe, there is a widespread tendency to reduce as much as possible the use of assays that involve the use of vertebrate organisms. Draft reports from EU REACH Implementation Project 3.3 recommend the use of vertebrates only if necessary. For example, in the aquatic ecotoxicity assessment, the sequence of testing involves the use of simple organisms for first test, and assays on fish only if necessary [3].

**Figure 4:**
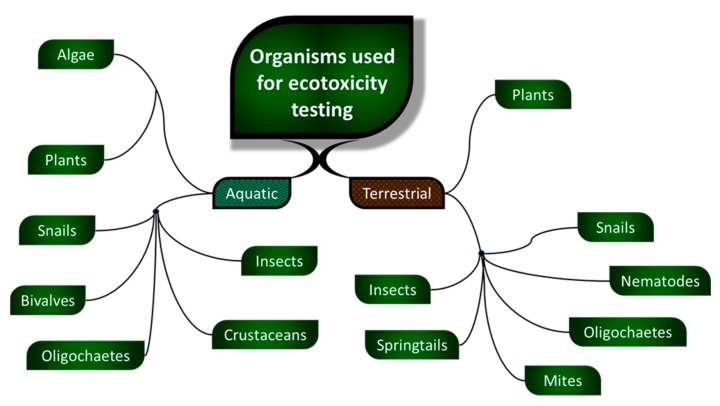
Categories of organisms that are used in standard guidelines for ecotoxicity assessment, several of the mentioned organisms being suitable for simple and rapid ecotoxicity testing (SSRET).

In conformity with this trend, in the present review, we used the term “SSRET organisms” for organisms suitable for simple and rapid ecotoxicity testing. We considered as SSRET organisms those organisms that are superior not from an evolutionary, taxonomical or complexity point of view, but from the point of view of suitability for ecotoxicity assessment. This term was assigned to species of plants (algae and vascular plants) and animals (only invertebrates) that are suitable due to their mechanisms of reproduction, size, culture method and also the lack of a proper ethical regulation, including only species that are not on the International Union for Conservation of Nature (IUCN) Red List [5] or protected by law. Only ecotoxicity assays that use SSRET organisms were studied because these give advantages like short culture period, are cheaper to maintain, need smaller laboratory space, etc. The organisms used in standard guidelines for ecotoxicity testing also include SSRET organisms from various categories such as those described in Figure 4.

The assays that use vertebrates and other unsuitable animal species, including fish, amphibians, reptiles, birds and mammals, that are subjected to ethical regulations and that are difficult to use in ecotoxicity assays due to their larger size, longer reproduction cycles and difficult culture methods, were excluded from our study. The assays conducted on vertebrates, even though they are needed in some cases, present disadvantages like ethical problems, expensive culture methods, long testing periods.

### 1.2. Nanomaterials

“Nanomaterial” (NM) is a term that usually describes materials that have external dimensions or internal structures measured in nanoscale. The materials exhibit additional or different unique properties, than the same chemical substance in non-nano form (bulk material or pristine chemical), such as optical, magnetic, conductive, mechanical and often biological properties [6]. The definition of nanomaterials (NMs) according to the International Organization for Standardization (ISO) is: a nanomaterial is a material with any external dimension or internal structure or surface structure in the nanoscale (1–100 nm) [3].

In this study the term “nanomaterial” is addressed to any chemical or mixture resulted after a chemical reaction or a physical process and which have at least one dimension in nanorange. Anthropogenic bionanomolecules are named bionanomaterials (BNMs) and natural biomacromolecules (polymers such as proteins, polysaccharides, nucleic acids), which naturally possess at least one nanodimension, are not named nanomaterials but pristine bionanomolecules (PBNMs).

To properly understand and appreciate the diversity of NMs, some form of classification is required:

- by dimension
  ○ zero-dimensional (0D)
  ○ one-dimensional (1D)
  ○ two-dimensional (2D)
  ○ three-dimensional (3D)
- by composition
  ○ carbon based NMs
  ○ organic based NMs
  ○ inorganic-based NMs
    ▪ metal-based NMs
    ▪ metal oxide NMs
  ○ composite-based NMs [6]

Nanomaterials with all dimensions (x, y, z) less than 100 nm and length equal to width are zero-dimensional nanoparticles (0D-NPs). This class of NMs includes mostly spherical materials, but cubes and polygonal shapes can also be found. Types of 0D-NPs are: molecules, particles, atomic clusters and grains, for example silver and gold nanoparticles and quantum dots [6,7].

Nanomaterials with two dimensions (x, y) less than 100 nm and one dimension beyond the nanoscale (> 100 nm) are 1D-NMs. Examples of 1D-NMs are nanotubes, nanowires, nanofilaments, nanofibers, nanorods, nanowhiskers, etc. [6,7].

The materials that have three arbitrary dimensions beyond the nanoscale (> 100 nm) but have a nanocrystalline structure or involve the presence of features at the nanoscale are 3D-NMs. Examples of 3D-NMs include bulk materials composed of individual blocks, such as skeletons of fibers and nanotubes, honeycombs, composites of layers, fullerites, etc. [6,7].

Nanomaterials can be classified into two main categories: natural NMs, which existed in the environment long before the nanotechnology era started, and anthropogenic NMs. Airborne nanocrystals of sea salts, soil colloids, fullerenes, carbon nanotubes, biogenic magnetite etc. are some examples of natural NMs. Anthropogenic NMs can be further divided into two categories: incidental and engineered/manufactured. Incidental NMs are produced unintentionally in manmade processes (e.g., carbon nanotubes, carbon black and fullerenes, platinum and rhodium-containing nanoparticles from combustion byproducts). Engineered/manufactured NMs are materials that are produced intentionally due to their nanospecific properties [8].

**Figure 5:**
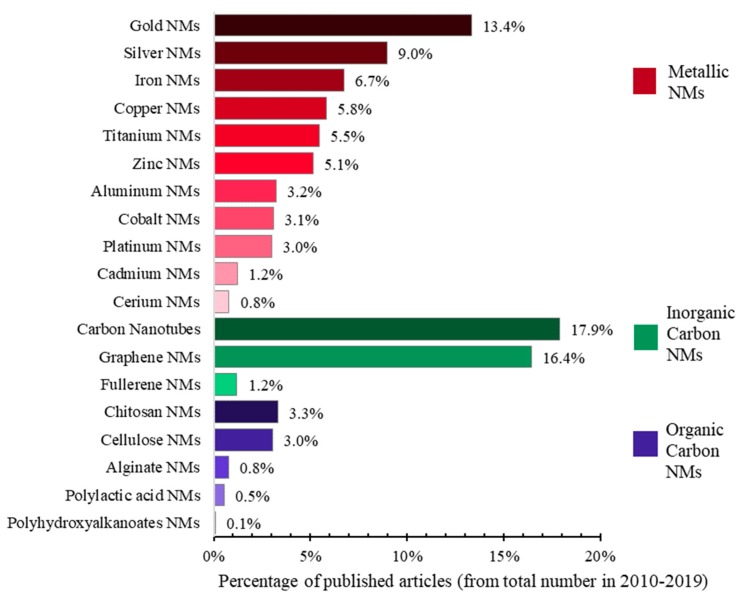
Bibliometric analysis of research on various types of nanomaterials in 2010–2019 from Web of Science Core Collection ( Web of Science, Clarivate Analytics), the search strategy used to retrieve the data being: TS=(Nano* AND polyhydroxyalk*), TS=(Nano* AND polylact*), TS=(Nano* AND algin*), TS=(Nano* AND cellulos*), TS=(Nano* AND Chitosan*) TS=(Nano* AND fuller*), TS=(Nano* AND graphen*), TS=(Nanotub* AND carbon*), TS=(Nano* AND cerium*), TS=(Nano* AND cadmium*), TS=(Nano* AND platinum*), TS=(Nano* AND cobalt*), TS=(Nano* AND aluminum*), TS=(Nano* AND zinc*), TS=(Nano* AND titanium*), TS=(Nano* AND copper*), TS=(Nano* AND iron*), TS=(Nano* AND silver*) and TS=(Nano* AND gold*).

The largest percentage of scientific articles on nanomaterials from the Web of Science Core Collection (Web of Science, Clarivate Analytics) in 2010–2019 is observed in the case of carbon nanotubes, followed by fullerene-based nanomaterials and those from gold and silver. The least investigated are the nanomaterials from polyhydroxyalkanoates, polylactic acid, alginate and cerium (Figure 5).

Nanotechnology is an interdisciplinary field with enormous potential in the fields of clean energy, medicine, chemistry, physics, nano-electronics, agriculture, astronomy, and environmental remediation. The physical, chemical, biological, catalytic and optical properties of materials become different from their bulk counterparts when their size becomes nanoscale (1–100 nm) [9].

## 2. Ecotoxicity Assessment of Nanomaterials on SSRET Organisms

In recent years the manufacturing, production and use of engineered nanomaterials (ENMs) in a wide range of products has increased. Releases of ENMs may occur during the use of nano-enabled consumer and industrial products either by nonintentional releases, like weathering of products containing NMs, intentional releases, like NMs used for environmental remediation, or accidental releases, like accidental spills during production, transportation or disposal [10].

There is a trend to include the ecotoxicity tests as part of the safe-by-design concept of creation, fabrication, utilization and disposal of ENMs.

Ecotoxicity assays can be either acute (short-term) or chronic (long-term). Acute assays are the most common measurement of ecotoxicological effects and are frequently used first to evaluate the survival of the organisms. Chronic tests are used when results from short-term tests combined with large safety factors suggest that there may be risks to the environment. These assays evaluate the sublethal effects on organism growth or reproduction [3].

The assessment of ecotoxicological effects of previously untested substances, as in the case of nanomaterials, is a challenging task. NMs are very rarely tested as potential pollutants, in contrast with their large diffusion. The main difficulties in assessing toxicity are a consequence of their colloidal nature and dynamics, as systems in which smaller or larger aggregates can form in poorly predictable ways, making it difficult to measure shape, size and concentration in the final sample. A basic requirement for good practice in toxicology laboratories is the use of approved or certified standards, which seems far from fulfillment in the case of NMs. The organization of the dispersed nanophase, in all naturally occurring systems like water, soil, air and their combinations, depends equally from the physical chemistry of the ENMs and from that of the environment, as well as from the modalities of suspension [11].

The assays selected for the ecotoxicity test battery for the assessment of the ecotoxicological effects of nanomaterials must be suitable for NMs because these materials present specific properties, different from their bulk materials, which may not be compatible with the assessment methods from the standard guidelines. There are several aspects in which NMs could interfere with the testing methods such as:

- agglomeration in test media during the procedures of test suspensions preparations;
- agglomeration, dissolution, and association with dissolved chemical species and colloidal/particulate matter already present in the natural waters;
- sedimentation due to agglomeration in the water column;
- chemical transformation processes such as sulphidation, hydroxylation;
- adhesion/deposition of nanomaterial onto soil minerals [10].

To our knowledge, there are not yet published articles or other documents stating that one or more of the standardized ecotoxicity assays are not suitable for NMs. Furthermore, the conclusion of the OECD Working Party on Manufactured Nanomaterials (WPMN) was that, in principle, the test guidelines are considered to be suitable also for the testing of NMs, although some adaptations were found to be necessary [12].

**Figure 6:**
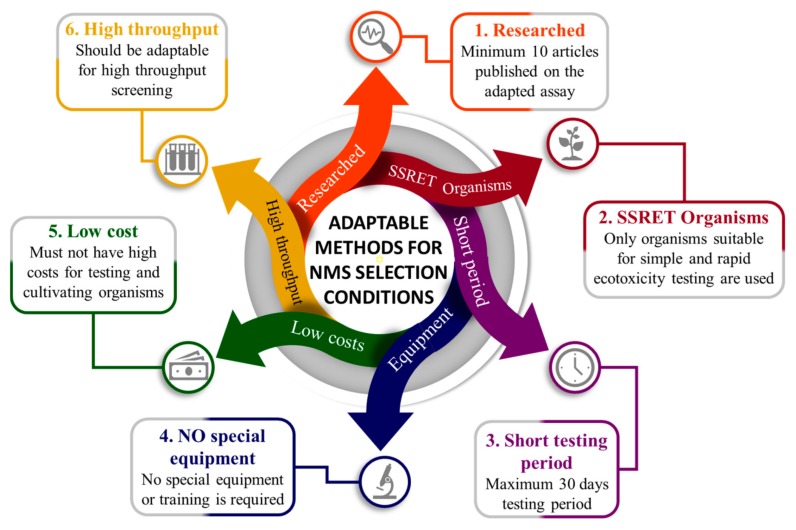
Condition for the selection of standard methods for ecotoxicity assessment that can be adapted for nanomaterial.

Since the assessment of ecotoxicity of NMs is imperative, the already developed and standardized assays must be sorted and selected, in order to create an efficient and adapted ecotoxicity test battery for NMs. Thus, we considered that an assay can be used to test NMs if the following conditions (Figure 6) are met:

- minimum 10 articles have already been published with the assay applied and adapted to NMs;
- only SSRET organisms are used;
- assessment period not longer than 30 days;
- no special apparatus or training needed;
- low testing costs;
- high-throughput methods (if possible).

Even if the majority of the standard tests could be adaptable, we only considered those that have already been published in minimum 10 scientific articles in which the assay was applied and adapted to NMs. We chose this rule in order to ensure that the theoretical adaptability is proven by research results. 

Assays that have testing periods longer than 30 days were excluded because the ecotoxicity test battery’s purpose is to make the testing easier and more relevant. Also, the companies that produce NMs or products that contain NMs, want to test their materials as fast as they can to ensure their profit, thus they cannot wait long periods of time for the results.

The use of special apparatus and/or special training increases testing time by forcing the companies to search for laboratories that have these specific apparatus and staff. These kinds of assays can also increase the cost of testing since special reagents might be needed. The cost of the assessment is important too, since the profit of a company that produce NMs or products that contain NMs depends on the cost of testing. 

An ideal ecotoxicity test battery should be easy and fast to apply, with specific tests for the selected material, and, in this case NMs, give relevant data for the material’s safety for the environment. From this point of view, high-throughput assays would be the best choice since these minimize the time of the test, by conducting several tests at once, and reducing the quantities of reagents needed, thus lowering the costs too. High-throughput screening (HTS) is an alternative technique to the “classical” method for scientific experimentation that allows a researcher to quickly conduct several tests concomitantly, by using, for example, a microtiter plate instead of test tubes.

An ideal ecotoxicity test should be predictive (the effect of NMs should be predictable outside the range of tested concentration) and transferable (the effect of NMs should be the same or very similar on non-tested organisms). 

From the tests listed in Table 4, which contains assays with testing time less than 30 days, only the tests that are compatible or adaptable for nanomaterials are selected and described. If a test is widely used for nanomaterials (minimum 10 articles published) but the majority of the described adaptations are only for the NM suspension preparation, it is still described if at least a few test adaptations are mentioned. Also, the tests that cannot be made without special apparatus or training were eliminated from the descriptions. The tests that were eliminated are listed below:

- the watermilfoil assays (sediment free toxicity assay and water-sediment toxicity assay) were eliminated due to the restricted number of published articles; in the published articles there are presented only the modification for the preparation of NM suspension, but not for the assay itself;
- the snail assays (on mud snail and on pond snail) were eliminated due to the restricted number of published articles, because bioconcentration was primarily assessed which takes longer than 30 days and because it involves the use of special apparatus like a flame atomic absorption spectrometer or MC-ICP-MS (Multicollector-Inductively Coupled Plasma Mass Spectrometer);
- the bivalve, aquatic oligochaetes, mysid, penaeid, enchytraeid, mite, springtail and the bumblebee assays were eliminated due to the restricted number of published articles; the published articles present only the modification for the preparation of NM suspension, but not for the assay itself;
- the fly assay was eliminated because only a few articles were found for ecotoxicity assessment of NMs; even if there are numerous ecotoxicity articles for NMs using as test organism *Drosophila sp*., these assays were not included in the description due to a lack of standardization.

**Figure 7:**
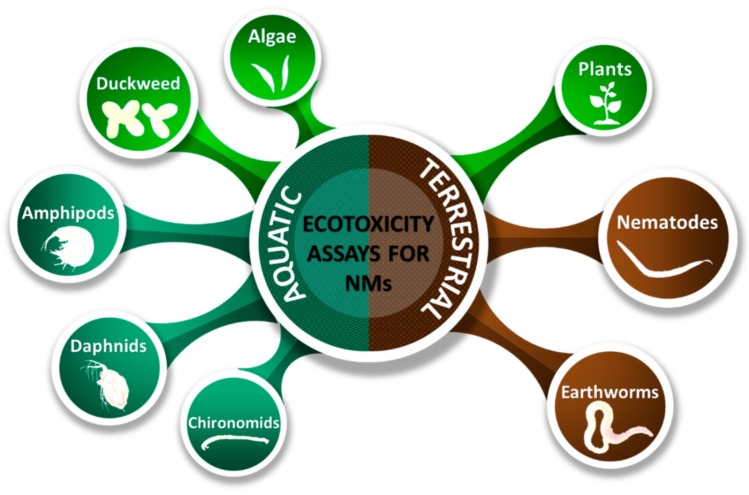
Standard assays selected to be adaptable for NMs.

Only eight standard assays were selected as being adaptable for NMs, five aquatic assays and three terrestrial ones: the algae, duckweed, amphipods, daphnids, chironomids, terrestrial plants, nematodes and earthworm assay (Figure 7).

## 3. Description of Ecotoxicity Assays Suitable for Nanomaterials and the Necessary Adaptations

### 3.1. General Adaptations and Considerations

Multiple factors need to be taken into consideration when bioassays involving exposure to suspended NMs are interpreted, such as: the relevance and the appropriateness for assessing the tested NMs; the accuracy of the representation of the exposure, e.g., whether the frequency of characterization measurements sufficient to capture changes in exposure during the bioassay; the consistency of the exposure, such as stable concentration, agglomeration and dissolution; whether maintaining a consistent exposure is possible in the bioassay method-specific test system; whether nanospecific bioassay acceptability criteria, e.g., sufficiently consistent exposure concentration with respect to agglomeration and dissolution, are met; and whether the characterization and monitoring data during the bioassay are amenable to expressing data by an alternative dose metric [13].

#### 3.1.1. Physico-Chemical Adaptations and Considerations

A wide range of dispersing techniques is used (application of solvents, dispersion or stabilizing agents, bath or probe sonication, stirring, etc.) and the methods also vary with respect to applied concentrations, time, etc. The NMs could be significantly altered by the preparation method, the used solvents could interact producing toxic byproducts and the properties of the dispersions depend on the dispersing method, and thus the ecotoxic effects of the NMs could be influenced and the comparability between tests could be hampered [14].

As described by previous OECD documents for metal toxicity testing, such as algae testing, it is important to exclude metal chelators such as ethylenediaminetetraacetic acid (EDTA) for NMs where dissolved metal ions may impact the toxicity (e.g., ZnO ang Ag NPs). The interactions between the chelators and the NM surface may influence the NM behaviors and transformations [13].

#### 3.1.2. Biological Adaptations and Considerations

It is important to ensure that only the portion of the contaminant that has actually accumulated is considered when bioaccumulation, bioconcentration, bioamplification and trophic transfer factor (TTF) are assessed in ecotoxicity studies. Only contaminants that are absorbed inside the organism must be considered. The contaminants should not be considered if these are on the surface of the organism or if these are ingested but can be eliminated by simple excretion. Thus, NMs that are trapped inside the digestive tract and prevent normal exchanges such as nutrient uptake, which can result in physiological impairments, should be considered as accumulated. To correctly consider a NM accumulated in the digestive tract, the organisms should be rinsed and allowed to depurate (i.e., they should be placed in a non-contaminated environment long enough for complete excretion to occur) so that only the absorbed contaminant is left in the organism. Absorption of NMs by the organism can result from several processes such as direct entrance in the cell (e.g., intestinal, skin or gill cells) or crossing of epithelial barriers through intercellular junctions to enter the blood circulation and then be distributed to the whole organism, without entering the cells [15].

### 3.2. Aquatic Plants Used for Ecotoxicity Testing of Nanomaterials

#### 3.2.1. Algae 

These organisms are used in ecotoxicity assessment through growth inhibition and toxicity assays. These assays are regulated by several standard guidelines created by organizations like OECD [16], EPA [17,18] and ISO [19,20].

**Figure 8:**
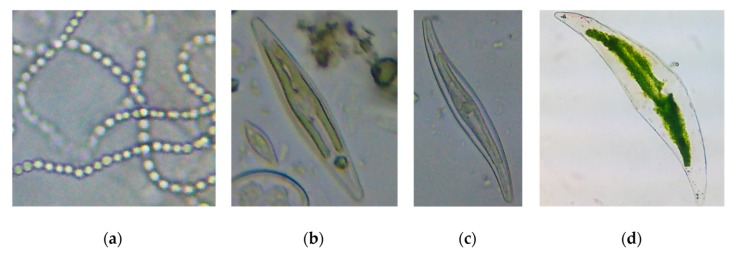
Examples of algae that can be used for ecotoxicity assessment: (**a**) cyanobacteria; (**b**) diatoms and (**c,d**) green algae.

The purpose of these assays is the determination of the ecotoxic effects of a substance on the growth of freshwater microalgae and/or cyanobacteria (Figure 8). Test organisms that are in the exponential growth phase are exposed to the test substances over a period of 72 hours (during which effects over several generations can be assessed), which can be extended to 96 hours for the assessment of toxicity by obtaining population growth data such as cell density [16,17].

- Adaptations
  ○ an EDTA-free version of the OECD medium for *Raphidocelis subcapitata* is recommended for metallic NMs [12];
  ○ by supplying iron as FeSO_4_ instead of FeCl_3_, the OECD medium permits a better growth for the algae. With this medium the amount of phosphorus is also increased, as mono and dibasic salts [12];
  ○ a shacking procedure is recommended during the tests [21];
  ○ the determination of algal biomass by cell counting with a hemocytometer is very laborious, has a large variance and it may not reflect the true biomass if there are changes in the mean size of the cells as a response to a toxicant or other condition. The interference caused by NMs could be avoided by the determination of biomass by fluorometry followed by *Chl a* extraction [12];
  ○ the determination of in vivo chlorophyll content could be realized in microtiter plates but NMs might interfere with the measurements [21];
  ○ the in vivo determination of fluorescence was found to be an unstable parameter; it depends on the prior light exposure of the culture, since different results are obtained by the repeated measurement of chlorophyll on the same sample [12];
  ○ the most reliable quantification method of algal biomass for testing the effects of NMs has been found to be the measurement of fluorescence of chlorophyll extracts. However the background fluorescence of the NMs should be reduced as much as possible [21].
- Uptake
  ○ The uptake of NMs in bacteria or algae can’t take place without the attraction of the NMs to a biofilm or their absorption to a substrate. The attachment of NMs to the cell membrane is assumed to take place via electrostatic attraction, but there are other forces that could be involved such as random collision. If no uptake is observed in microalgae it is possible that the concentration of NMs was too low for attachment by collision to occur or that the electrostatic attraction was too weak for adsorption to have taken place [22].
  ○ Released metal ions from metallic NMs may diffuse across cell membranes. For some cyanobacteria (e.g., *Anabaena flos-aquae*), however, by the production of extracellular polysaccharides the internalization of NMs could be avoided due to the electrostatic interaction of these substances with NMs, which are entrapped outside the cell [22].
- Advantages
  ○ short testing period of 72–96 hours;
  ○ high-throughput assay microplates can be used;
  ○ simple culture method;
  ○ simple quantification method: chlorophyll extraction;
  ○ one of the most sensitive ecotoxicological assays [23].
- Disadvantages
  ○ fluorescence reader is necessary for optimal chlorophyll quantification;
  ○ NM–algae aggregates may form: heteroaggregation of NM-algae was reported [21];
  ○ NMs may cause shading by scattering the light from reaching algal cells and thereby reduce their growth rate, rather than or in addition to any toxic effect. Even if the algae can temporarily overcome the shading from distant NMs, the adhesion of NMs to the algal cells can result in permanent shading and limitation of nutrient availability [24];
  ○ if optical density measurements are used, there could be interferences with the NMs [23].

#### 3.2.2. Duckweed 

**Figure 9:**
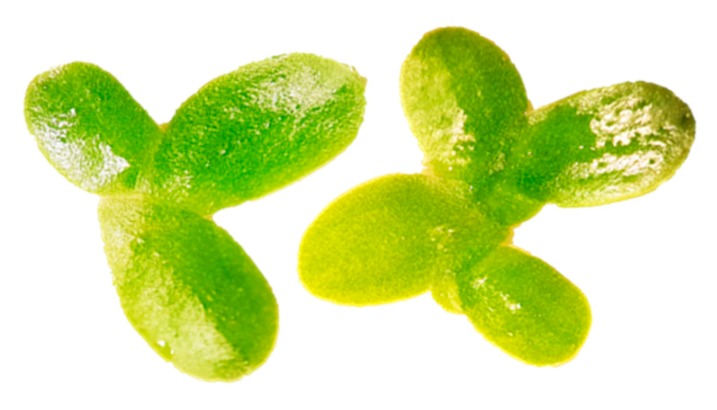
Example of duckweed species recommended for ecotoxicity assessment by standard guidelines: *Lemna minor* (common duckweed).

These organisms (Figure 9) are used in ecotoxicity assessment through growth inhibition and toxicity assays. These assays are regulated by several standard guidelines formulated by organizations like OECD [25], EPA [26] and ISO [27,28].

Exponentially growing duckweed cultures are grown as monocultures in test substances of different concentrations over a period of seven days. The quantification of substance-related effects on vegetative growth can be based on the assessment of different variables such as frond number, which is the primary variable of measurement, and at least one other (e.g., total frond area, fresh or dry weight, chlorophyll content). The endpoint of this assays is percent inhibition in average specific growth rate and 50 percent inhibition (IC_50_) [25].

- Adaptations
  ○ the careful stirring twice a day of the growth medium is recommended to minimize the sedimentation of NMs [29];
  ○ it is recommended to wash the plant samples using EDTA-Na_2_ (0.02 M, five times) and distilled water (five times) to avoid the possible attachment of metallic NMs to the samples [30].
- Advantages
  ○ simple cultivation method;
  ○ can be grown indefinitely as genetically homogeneous clonal colonies due to their predominantly vegetative reproduction [31];
  ○ ready contact with substances dissolved in the culture medium is ensured by their high surface-to-volume ratio and lack of cuticle on their surface in contact with water [31];
  ○ some species of duckweed have a wide pH tolerance such as *Spirodela polyrhiza* [32].
- Disadvantages
  ○ medium testing period: seven days;
  ○ relatively large laboratory space necessary for testing of multiple experiments at once;
  ○ the actual environmental conditions, under which the test organisms live in nature, are not accurately reflected by the standard experimental conditions employed in any standardized duckweed toxicity test [31].

### 3.3. Aquatic Invertebrates Used for Ecotoxicity Testing of Nanomaterials

#### 3.3.1. Amphipods

**Figure 10:**
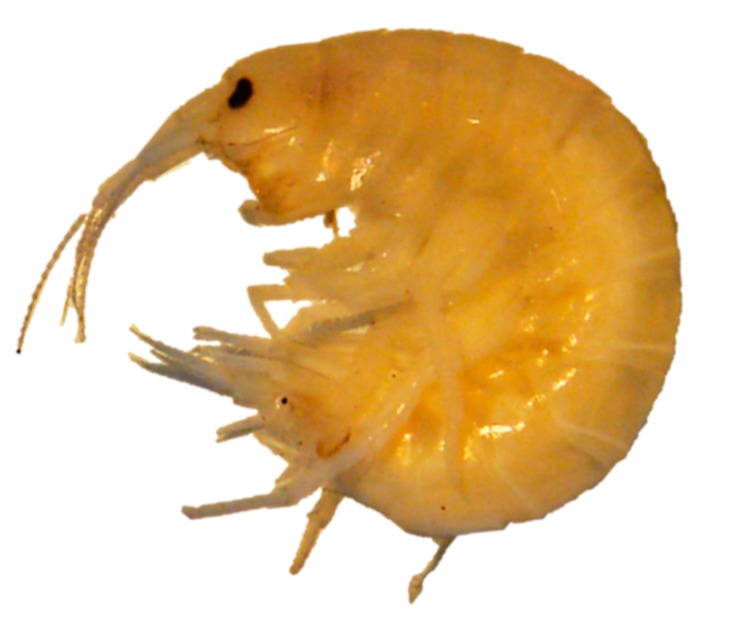
Example of amphipod species that can be used for ecotoxicity assessment: *Gammarus sp.*

These organisms (Figure 10) are used in ecotoxicity assessment through acute toxicity and spiked whole sediment toxicity assays. These assays are regulated by several standard guidelines formulated by organizations like EPA [33,34,35] and ISO [36].

In the acute toxicity assay, young gammarid amphipods are exposed to the test substance and to appropriate controls for 96 hours. Observations are made regarding the survival of the organisms and other toxic effects. The relationship between aqueous concentrations of the test substance and mortality of gammarids over the full concentration–response curve is determined by this assay [33].

The spiked whole sediment toxicity assay involves the monitoring of amphipods during the test for sediment avoidance and other toxic effects observable without disturbing the sediment. The survival and growth are determined at the termination of the test [34,35].

- Adaptations
  ○ only NM suspension preparation adaptations are specified.
- Advantages
  ○ short testing period for the acute toxicity assay: 96 hours.
- Disadvantages
  ○ relatively large laboratory space necessary for testing of multiple experiments at once.

#### 3.3.2. Daphnia

**Figure 11:**
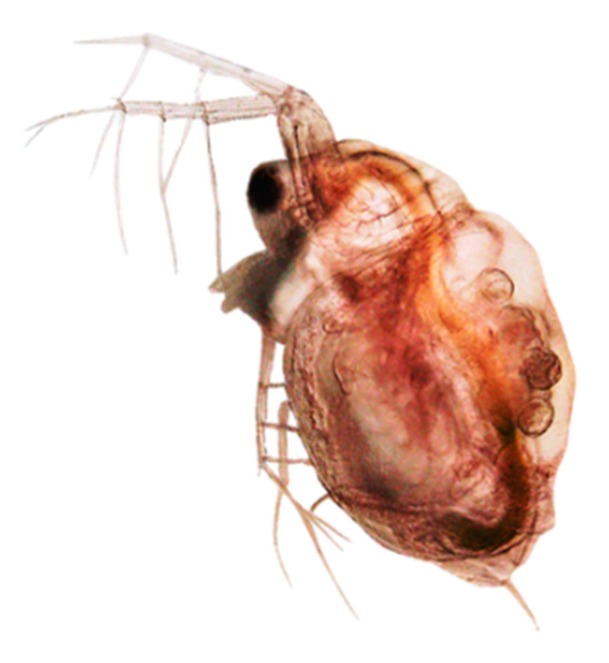
Example of daphnia species recommended for ecotoxicity assessment by standard guidelines: *Daphnia magna*.

These organisms (Figure 11) are used in ecotoxicity assessment through an acute toxicity (immobilization) assay or a reproduction assay. Both assays are regulated by several standard guidelines formulated by organizations like OECD [37,38], EPA [39,40] and ISO [41,42,43].

The acute toxicity assay involves the use of young daphnids (with age less than 24 hours at the start of the test) and their exposure to the test substance at a range of concentrations for a period of 48 hours. The endpoint of this assay is immobilization (lack of motility) of daphnids, which is recorded at 24 hours and at 48 hours and compared with control values. The half maximal effective concentration at 48 hours is calculated by analyzing the obtained results [37].

In the reproduction assay young female daphnids (aged less than 24 hours) are exposed to the test substance (at a range of concentrations) added to water. The assessment of the effect of chemicals on the reproductive output of daphnids is the primary objective of this assay. At the end of the test, after 21 days, the total number of living descendants produced is determined. Other ways can be also used for the expression of the reproductive output of the parent animals but these should be reported in addition to the total number of living offspring produced at the end of the test [38].

NMs or NM-algae associations could be consumed by *Daphnia magna*. The water with the NMs is funneled towards the daphnia’s mouth by its feeding appendages. In the gut lumen, the NMs are accumulated after being compacted by the undigested food and other materials. The accumulation of NMs in the gut lumen and their condensation into a tightly packed form was also observed in marine copepods such as *Tigriopus japonicus* [22].

- Adaptations
  ○ the use of a medium with a very low ionic strength, under which daphnids can grow and reproduce normally, and which has a pH value where more stable dispersions can be obtained is recommended [12];
  ○ NMs can be absorbed on the exoskeleton, cuticle and antenna of crustaceans like daphnids. This influences the mobility, molting and swimming velocity of the tested crustaceans. Thus, the inclusion of both lethality and immobilization assays is recommended. The use of only immobility as endpoint, such as the OECD assay, may be problematic in cases where immobility reflects physical impairment rather than toxicity [24];
  ○ to prevent the mechanical impairment of daphnids by absorbed NMs, a mesh could be inserted at the bottom of the test vessel to prevent the contact of the daphnids with larger clusters of NMs that accumulate at the bottom of the beaker [24];
  ○ greater water hardness is used for *Daphnia magna* growth and reproduction assays which leads to a greater agglomeration rate of NMs for charge-stabilized NMs, resulting in less consistent exposure. Thus, using daphnid species that are adapted for softer waters is recommended (such as *Daphnia pulex*) [13];
  ○ to increase the contact between the daphnids and the NMs the use of shallow test vessels or semi-static and flow-through systems is recommended [21];
  ○ the feeding of daphnids during the reproduction assay is not recommended. It was observed that the outcome of the test is food quantity dependent, as the addition of higher food levels resulted in higher animal survival, growth and reproduction compared to tests with lower food levels. Also, the uptake of NMs is influenced by the presence of food. This may influence the chronic effects found in long-term exposure tests. Furthermore, the presence of food (algae) in daphnia reproduction test may affect the observed toxicity, due to the interaction of NMs with algal exudates which affect the bioavailability of the NMs [24].
- Advantages
  ○ short testing period for acute toxicity assay: 48 hours;
  ○ daphnia are particle-feeding organisms, a relevant model for NMs [23];
  ○ daphnids are one of the most sensitive organisms used in ecotoxicity assessment of chemicals [37].
- Disadvantages
  ○ long testing period for reproduction and chronic assays: 21 days;
  ○ high sample volume is required (50 mL per concentration) [23];
  ○ test medium may induce agglomeration and sedimentation of NMs [44];
  ○ NMs may affect the movement of the daphnids by provoking physical effects on their surface [21].

#### 3.3.3. Chironomids

**Figure 12:**
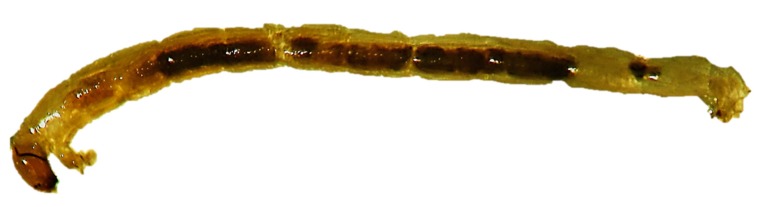
Example of chironomid that can be used for ecotoxicity assessment.

These organisms (Figure 12) are used in ecotoxicity assessment through a sediment water toxicity assay or a sediment water life cycle toxicity assay, using either spiked sediment or spiked water or an acute immobilization assay. These assays are regulated by several standard guidelines formulated by organizations like OECD [45,46,47,48] and EPA [34].

The sediment water toxicity assay with spiked sediment involves the exposure of first instar chironomid larvae to a concentration range of the test chemical in sediment–water systems, where the test substance is spiked into the sediment. The first instar larvae are subsequently introduced into test beakers with stabilized sediment and water concentrations. At the end of the test the emergence and development rate of chironomids are measured. If required, after ten days, the larval survival and weight can be also measured [47].

The sediment water toxicity assay with spiked water involves the exposure of first instar chironomid larvae to a concentration range of the test chemical in sediment–water systems, where the test substance is spiked into the water. At the end of the test the emergence and development rate of chironomids are measured [48].

The sediment water life cycle toxicity assay with spiked water or sediment involves the exposure of first instar chironomid larvae to a concentration range of the test substance in a sediment-water system. The first instar larvae (first generation) are placed into test beakers that contain the test substance spiked into the sediment or water. At the end of the test the chironomid emergence, time to emergence and sex ratio of the fully emerged and living midges are assessed. To facilitate the swarming, mating and oviposition of the emerged adults, these are transferred to breeding cages and the number of egg ropes produced is assessed. The second generation first instar larvae obtained from these egg ropes are then placed into freshly prepared test beakers to determine their viability through an assessment of their emergence, time to emergence and the sex ratio of the fully emerged and living midges [46].

The acute immobilization assay involves the exposure of first instar *Chironomus sp.* larvae to a range of concentrations of the test substance in water-only vessels for a period of 48 hours. The immobilization of the chironomids is recorded both at 24 hours and at the end of the test, at 48 hours [45].

- Adaptations
  ○ an additional parameter is recommended for the assessment of the toxic effects. This parameter is represented by morphological malfunctions revealed by the analysis of the mouthpart structures [49].
- Advantages
  ○ short testing period for acute tests: 48 hours;
  ○ chironomids have a widespread distribution. The aquatic sediment environment is strongly influenced by them through processes such as sediment ingestion, digestion, resuspension, excretion, and secretion, bioirrigation and bioturbation [50].
- Disadvantages
  ○ long testing periods for the sediment–water system tests: 10–28 days.

### 3.4. Terrestrial Plants Used for Ecotoxicity Testing of Nanomaterials

**Figure 13:**
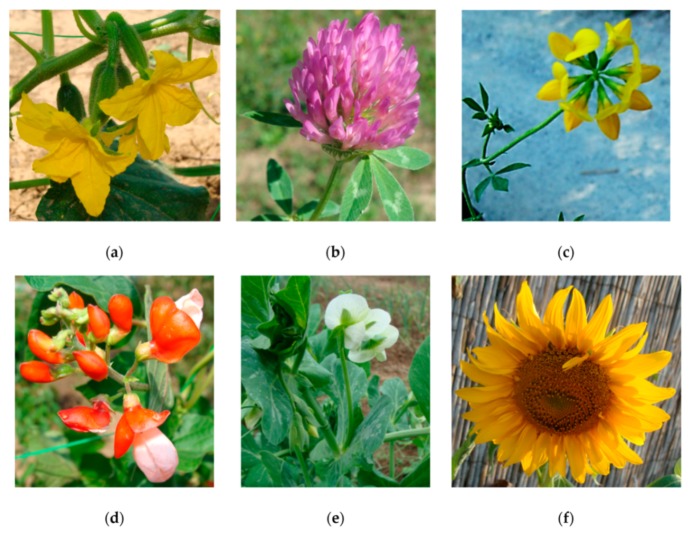
Examples of terrestrial plant species recommended for ecotoxicity assessment by standard guidelines: (**a**) *Cucumis sativus* (cucumber); (**b**) *Trifolium pratense* (red clover); (**c**) *Lotus corniculatus* (common birdsfoot trefoil); (**d**) *Phaseolus vulgaris* (common bean); (**e**) *Pisum sativum* (pea) and (**f**) *Helianthus annuus* (sunflower).

These organisms (Figure 13) are used in ecotoxicity assessment through an early seedling growth toxicity, a seedling emergence and seedling growth assay and a vegetative vigor assay. These assays are regulated by several standard guidelines formulated by organizations like OECD [51,52], EPA [53,54,55] and ISO [56,57,58,59,60].

The effect of a test substance applied to the roots or the leaves of several terrestrial plant species is assessed thorough the early seedling growth toxicity assay. After germination of seeds planted in the potting containers, seedlings are thinned (by pinching the stem at the support medium surface) to the 10 most uniform seedlings per pot to which the test substances are applied. The application of the test substances can produce either a foliar exposure (by exposing the plants in a fumigation chamber to gas or by spraying or dusting the foliage) or a root exposure (by nutrient solution or by sorption to support media). Seedlings emerging after this time are also pinched off at the surface. After 14 days, the plants are harvested, and their growth is analyzed by parameters such as observed phytotoxicity, length or dry weigh of the shoot or the root, seedling survival, length and weight of whole plants [55].

The effects of a test substance dosed to soil are assessed on seedling emergence and early growth of higher plants. The effects of the test substance on seeds are assessed after 14–21 days, after 50% emergence of the seedlings in the control group. Visual assessment of seedling emergence, dry or fresh shoot weight or height and visible detrimental effects on different parts of the plant are the measured endpoint of this assays, which are compared to those of untreated control plants [51].

Following deposition of the test substance on the leaves and above-ground portions of plants, the potential effects of those test substances are assessed through the vegetative vigor assay. The test substance is sprayed on plants, grown from seed to the 2–4 leaf stage, and their leaf surfaces at different concentrations. At various intervals through 21–28 days from treatment, the plants are evaluated for effects on vigor and growth against untreated control. Shoot dry or fresh weight and height, and visible detrimental effects on different parts of the plant are the assessed endpoints of this test [52].

- Adaptations
  ○ to avoid NMs precipitation, which are poorly soluble in water, and to distribute NMs evenly, the use of plant agar tests is recommended. In the preparation of the agar solutions, to avoid the possible precipitation of NMs, the test plates were immediately hardened after pouring in a freezer [61].
- Advantages
  ○ plants are critical to the function of ecosystems and the integrity of the food supply, thus plants being an essential component of the environment [62];
  ○ NMs are of an extensive interest for application on plants for uses in agriculture and horticulture [63].
- Disadvantages
  ○ long testing periods: 14–28 days;
  ○ the analysis of NM content of plants may imply the use of special apparatus like ICP-MS [61];
  ○ special techniques like the scanning electron microscopy–cathodoluminescence technique may be used for the analysis of NM content of agar paste [61];
  ○ the biological effects determined could depend on the species selected for the assay [64].

### 3.5. Terrestrial Invertebrates Used for Ecotoxicity Testing of Nanomaterials

#### 3.5.1. Nematodes

**Figure 14:**
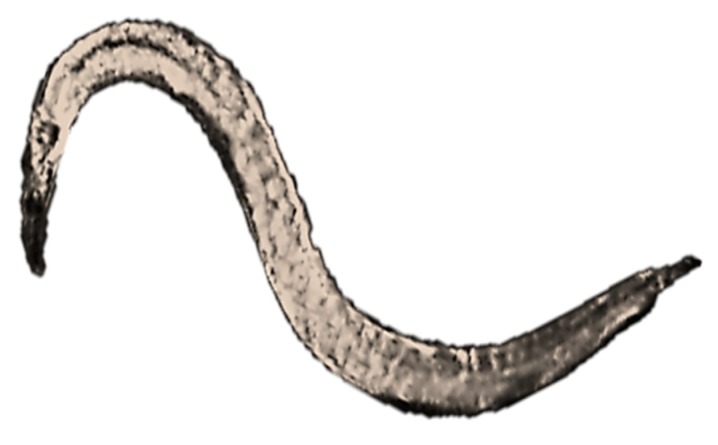
Example of nematode that can be used for ecotoxicity assessment.

These organisms (Figure 14) are used in ecotoxicity assessment through a soil and sediment toxicity test on growth, fertility and reproduction of *Caenorhabditis elegans*. This assay is regulated by several standard guidelines formulated by organizations like ISO [65] and ASTM [66].

The soil and sediment toxicity test on growth, fertility and reproduction of *Caenorhabditis elegans* involves the maintenance of nematode stock cultures grown on agar medium. For the aqueous assay, the stock solutions are transferred to 12-well microplates, while for the soil and sediment assays, the dry material is placed into test vessels and moistened with medium (to achieve 40% water content for the artificial control sediment and the soil samples and original water content for natural sediment samples). *Escherichia coli* suspended in medium is then mixed into the aqueous, sediment, and soil samples as food supply. At the end of the test, after 96 hours, the worms are heat-killed and then mixed with an aqueous solution of rose Bengal to stain them for easier counting [67].

- Adaptations
  ○ the NM suspension can be prepared by sonication [68];
  ○ during the test, the testing plates can be shaken [68].
- Advantages
  ○ short testing period: 96 hours;
  ○ can be applied in microplates, thus it is a high throughput assay [68];
  ○ a major change in the abundance of soil invertebrates such as nematodes, which are key organisms in soil, could have serious adverse effects on the entire terrestrial system [66];
  ○ due to its ability to grow and reproduce in both soil and aqueous environments, *Caenorhabditis elegans* is a well suited organism for toxicity assessment [69].
- Disadvantages
  ○ NMs can aggregate and may settle on the bottom of test vessels. This could potentially increase the local concentration and exposure to the NMs and could also change the exposure from NMs to NM aggregates [69];
  ○ the reproductive output could be decreased by the shacking of the test vessels [69];

#### 3.5.2. Earthworms

**Figure 15:**
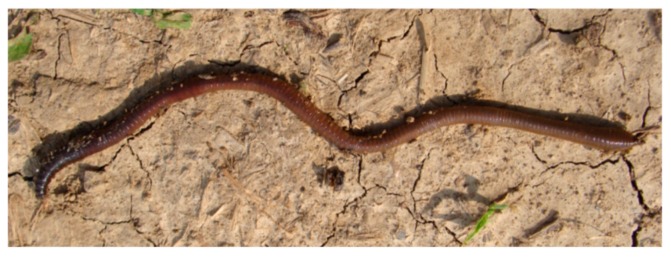
Example of earthworm that can be used for ecotoxicity assessment.

These organisms (Figure 15) are used in ecotoxicity assessment through an acute toxicity assay, and a subchronic toxicity assay. These assays are regulated by several standard guidelines formulated by organizations like OECD [70], EPA [71] and ISO [72].

The simple contact acute toxicity test involves the exposure of earthworms to test substances on moist filter paper to identify potentially toxic chemicals to earthworms in soil. This test gives reproducible results with the recommended species and it is easy to perform [70].

Data more representative of natural exposure of earthworms to chemicals can be obtained using the artificial soil acute toxicity test. In this test earthworms are kept in samples of a precisely defined artificial soil to which a range of concentrations of the test substance has been applied. Mortality is assessed seven and 14 days after application [70].

The subchronic toxicity assay involves the placement of acclimated worms in test chambers that contain artificial soil with the test substance. The earthworms ingest the soil mixture and at the end of the test, after 28 days, their mortality and other effects are assessed [71].

- Adaptations
  ○ the reduction of organic matter content of the standard OECD soil is recommended, due to the reduction of bioavailability of NMs by artificial soil (the NMs are absorbed to soil organic matter) [12];
  ○ it is recommended to dose the NMs by directly adding the dry nanopowder to the soil, because it was observed that NMs agglomerate in water at the concentration needed for the dosing of the soil [73];
  ○ to ensure a continuous exposure even if the worms would attempt to escape into the added food mixture, the food should also be dosed with NMs [73].
- Advantages
  ○ *Eisenia fetida* is a recommended species for ecotoxicity assays as it can be easily cultured in the laboratory [74];
  ○ earthworms represent 60–80% of the total soil biomass and have a wide range distribution of soil, thus being an ideal organism for use in ecotoxicity assays, as these are also sensitive organisms that are readily available. Furthermore, historical data are available for their use in ecotoxicity assessment [74].
- Disadvantages
  ○ long testing period: 14–28 days.

## 4. Ecotoxicity of Nanomaterials

Nanotechnology is slowly becoming an essential part of daily life in different forms, such as pharmaceuticals, food packaging, biosensors, cosmetics etc., and thus it might present an unintended risk to human health and the environment [9].

The environment could be polluted intentionally or accidentally by large quantities of NMs due to the increasing presence of these materials in commercial products. It is crucial that the potential environmental and health impacts of NMs are assessed and the environment is protected to ensure a sustainable nanotechnology industry [75].

**Figure 16:**
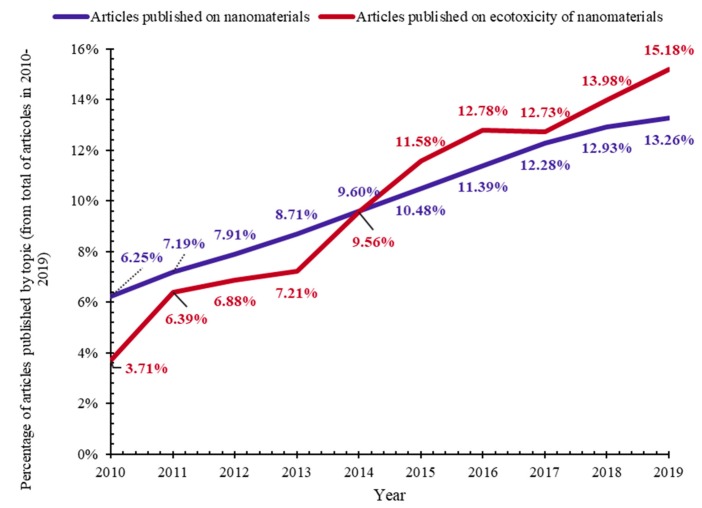
Bibliometric analysis of research on nanomaterials and their ecotoxicity from 2010 to 2019 from Web of Science Core Collection (Web of Science, Clarivate Analytics). The search strategy used to retrieve the data was TS=(Nano*) and TS=(Ecotox* AND Nano*).

Both the percentage of scientific articles on nanomaterials and the percentage of scientific articles on the ecotoxicity of nanomaterials from the Web of Science Core Collection (Web of Science, Clarivate Analytics) are increasing during 2010–2019 (Figure 16). This highlights directly the importance of testing the ecotoxicity of nanomaterials, but also, indirectly, the importance of adapting standard ecotoxicity tests to NMs.

**Figure 17:**
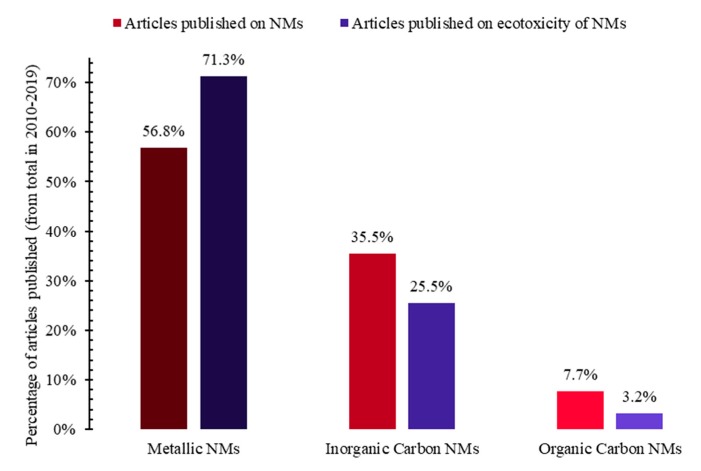
Bibliometric analysis of research on nanomaterials and their ecotoxicity by nanomaterial category in 2010–2019 from Web of Science Core Collection (Web of Science, Clarivate Analytics), the search strategy used to retrieve the data being: TS=(Nano* AND polyhydroxyalk*) AND TS=(Ecotox*), TS=(Nano* AND polylact*) AND TS=(Ecotox*), TS=(Nano* AND algin*) AND TS=(Ecotox*), TS=(Nano* AND aluminum*) AND TS=(Ecotox*), TS=(Nano* AND platinum*) AND TS=(Ecotox*), TS=(Nano* AND Chitosan*) AND TS=(Ecotox*), TS=(Nano* AND cobalt*) AND TS=(Ecotox*), TS=(Nano* AND cellulos*) AND TS=(Ecotox*), TS=(Nano* AND cerium*) AND TS=(Ecotox*), TS=(Nano* AND cadmium*) AND TS=(Ecotox*), TS=(Nano* AND graphen*) AND TS=(Ecotox*), TS=(Nano* AND fuller*) AND TS=(Ecotox*), TS=(Nano* AND silver*) AND TS=(Ecotox*), TS=(Nano* AND iron*) AND TS=(Ecotox*), TS=(Nano* AND gold*) AND TS=(Ecotox*), TS=(Nano* AND copper*) AND TS=(Ecotox*), TS=(Nano* AND zinc*) AND TS=(Ecotox*), TS=(Nanotub* AND carbon*) AND TS=(Ecotox*) and TS=(Nano* AND titanium*) AND TS=(Ecotox*).

Both the highest percentage of scientific articles on nanomaterials, as well as the highest percentage of scientific articles on the ecotoxicity of nanomaterials from the Web of Science Core Collection (Web of Science, Clarivate Analytics) can be observed in the case of metallic nanomaterials, followed by those of inorganic carbon. Only in the case of metallic nanomaterials, the percentage of scientific articles on the ecotoxicity of the nanomaterials was higher than the percentage of scientific articles on nanomaterials, percentages relative to the total scientific articles from 2010–2019 from the same topic (Figure 17). This highlights the necessity of scientific research on the ecotoxicity of organic carbon nanomaterials.

**Figure 18:**
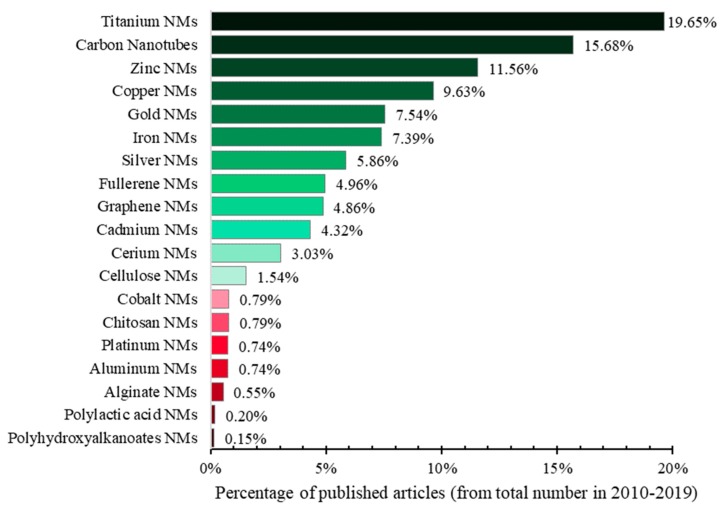
Bibliometric analysis of research on ecotoxicity of various types of nanomaterials in 2010–2019 from Web of Science Core Collection (Web of Science, Clarivate Analytics), the search strategy used to retrieve the data being: sum of the codes for NMs from each category (metallic NMs: aluminum, cadmium, cerium, cobalt, copper, gold, iron, platinum, silver, titanium and zinc; inorganic carbon NMs: carbon nanotubes, fullerene and graphene; organic carbon NMs: alginate, cellulose, chitosan, polyhydroxyalkanoates and polylactic acid).

The highest percentages of scientific articles on the ecotoxicity of different types of nanomaterials during 2010–2019 from the Web of Science Core Collection (Web of Science, Clarivate Analytics) can be observed in the case of titanium nanomaterials, carbon nanotubes and zinc nanomaterials. The lowest percentages can be observed in the case of nanomaterials of polyhydroxyalkanoates, polylactic acid, alginate, aluminum, platinum, chitosan and cobalt (Figure 18). This highlights the necessity of scientific research on the ecotoxicity of the least researched nanomaterials.

The conducting of risk assessment or the establishment of environmental quality standards and guidelines is difficult due to the poorly understood toxic effects of NMs, especially on wildlife [3].

The use of NMs depends on testing their safety prior to their application due to their harmful effects, even if nanotechnology also offers potential advantages and promises. The major hazards of toxicity of NMs lie in their chemistry, composition, surface properties, size, shape, solubility, nondegradable properties, routes of exposure, interactions with biological molecules, bioavailability, tissue distribution and property to accumulate in routes of entry, tissues and cells [76].

- Surface of NMs
  ○ control the distribution of materials in tissue;
  ○ NMs undergo adsorption on macromolecules of the tested organism;
  ○ ionic crystal NMs are observed to be accumulated in cytoplasm or body fluid through circulation.
- Size of NMs
  ○ controls the distribution and penetration of tissue by NMs;
  ○ reduction in size to nanoscale level leads to an increase of surface-to-volume ratio, thereby increasing the number of chemical molecules on surface, leading to an increase in intrinsic toxicity;
  ○ the NMs size can control the dose–response relationship in relation to its solubility and toxicity.
- Shape of NMs
  ○ plays an important role in determining the toxic nature of NMs, as high aspect ratio NMs (with only one or two dimensions in nanoscale) like nanofibers (that are longer than 10–20 µm and thinner than 3 µm) may remain in the pleural cavity, causing lung inflammation and even cancer (for example asbestos and carbon nanotubes).
- Aggregation of NMs
  ○ NMs tend to form aggregates;
  ○ the size of aggregates/agglomerates influences the residence time and reduces the potential for a NM to be inhaled;
  ○ the aggregation/agglomeration is controlled by external environment like air and dispersion media;
  ○ NMs may undergo disaggregation and disagglomeration within respiratory system, thereby penetrating lung cells [76].

The ecotoxicity of NMs is usually expressed by different concentration values such as EC_50_ (half maximal effective concentration), IC_50_ (half maximal inhibitory concentration), LOEC (lowest observed effect concentration), NOEC (no observed effect concentration), etc. To emphasize the ecotoxicity of these nanomaterials, biological and biochemical effects are presented on both SSRET organisms and vertebrates and other unsuitable (non SSRET) organisms, if data is available. Biological effects describe both anatomical and morphological aspects, as well as aspects related to body functions, such as locomotion and digestion. Biochemical aspects describe the mechanisms of action of these materials, such as interaction with metabolic pathways or certain molecules in the body, such as enzymes.

### 4.1. Ecotoxicity of Bionanomolecules

Both natural (pristine bionanomolecules) and engineered (bionanomaterials) bionanomolecules have a wide range of applications, which increases their likelihood of reaching the environment. Thus, as potential pollutants, it is necessary to test their ecotoxicity as well as to elucidate the mechanisms by which these nanomaterials affect the organisms.

Due to their applications in medicine and pharmacology, polymeric bionanomolecules, such as chitosan, alginate, poly lactic acid, etc., have been in focus in recent years. These polymeric BNMs can be synthetized by different methods, such as solvent evaporation, nanoprecipitation, interfacial polymerization and controlled gelation [77].

Although these bionanomolecules are mostly biocompatible and biodegradable, they can still have some ecotoxicological effects on some organisms. This is because biocompatibility tests are performed at lower concentrations, which certainly do not cause toxic effects. But the purpose of ecotoxicity tests is to determine the concentrations at which toxic effects occur, thus testing concentrations much higher than those that would be used in vivo or that would occur in the environment in normal conditions.

#### 4.1.1. Nanochitosan

**Figure 19:**
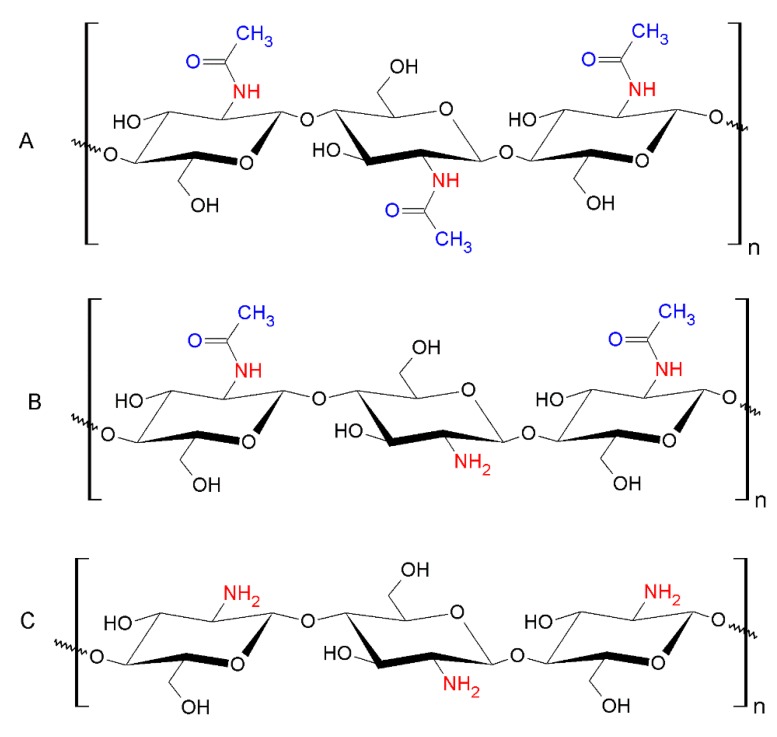
Structure of chitosan pristine bionanomolecules.

Chitosan, obtained by the deacetylation of chitin, is a polysaccharide that has two monomer units, N-acetyl-D-glucosamine and D-glucosamine (Figure 19). The many special properties of chitosan are due to its cationic nature and functional groups like amine and hydroxyl [78]. 

In nature, chitin, the source of chitosan, exists as long and straight microfibrils, that have an indeterminate length and a diameter of 2.8 nm [79]. These molecules (chitin and chitosan) may be considered as pristine bionanomolecules, as they have a dimension in nanoscale.

Chitosan bionanomaterials can be fabricated using different processes such as ionic gelation, covalent crosslinking or self-assembly. The most common forms of chitosan BNMs are nanogels, micelles, nanofibers, nanospheres and nanoparticles [80].

Chitosan BNMs and PBNMs are used as drug carriers, via various routes of administration such as oral, nasal, ocular or intravenous. The drugs these nano-chitosans could deliver could be variate, such as genes, proteins or antibiotics [81]. Another potential application is the encapsulation of vitamins, probiotics, phytochemicals, flavors, enzymes etc. [78]. In medicine, the most common applications are in tissue engineering, wound healing, cancer diagnosis, etc. [82].

The number of published articles aimed at determining the ecotoxicological effects of chitosan bionanomolecules is very low. Most published articles are in the field of medicine, chitosan BNMs being used for bone tissue regeneration and in wound healing. 

**Figure 20:**
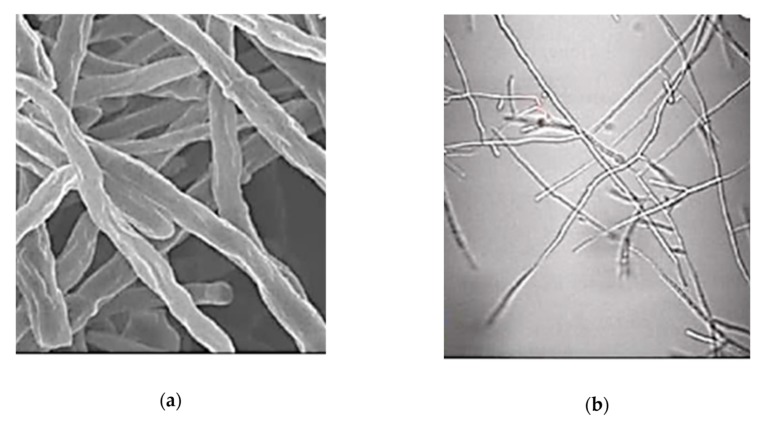

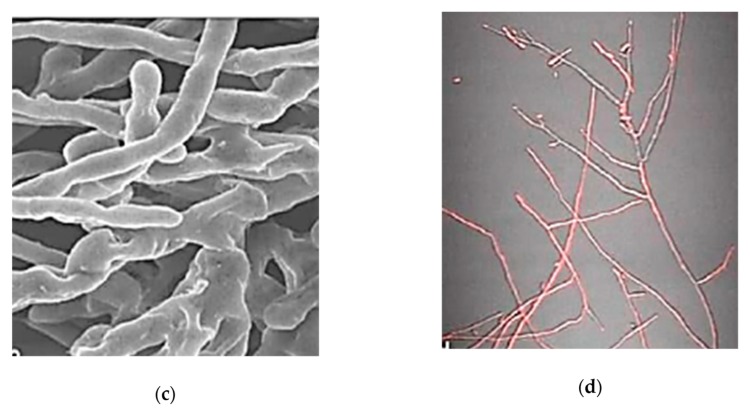
Comparison of ultrastructural and cell permeability changes in *F. oxysporum* mycelium upon chitosan bionanomaterial (BNM) exposure: (**a**) and (**c**) show ultrastructural changes determined by SEM analysis of *F. oxysporum* mycelium of control (**a**) and chitosan BNMs (400 µg/mL) (**c**); (**b**) and (**d**) show cell permeability analysis data by propidium iodide assay; (**b**) influx of control; (**d**) influx of chitosan BNMs treatment (modified after [84]).

**Figure 21:**
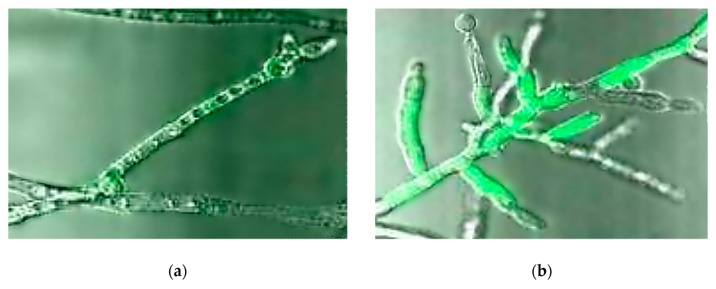
Confocal laser scanning microscopy (CLSM) image of *F. oxysporum* mycelium showing ROS production level upon exposure of chitosan BNMs. (**a**) control; (**b**) chitosan BNMs (400 µg/mL) ( modified after [84]).

The scarce data on ecotoxic effects show that chitosan BNMs have an antifungal effect on species like *Fusarium oxysporum*. The antifungal effects [83] include induction of morphological changes such as abnormal shapes of hyphae and large vesicles in mycelium, and induction of irreversible membrane damage, as well as mycelium surface damage, appearing as ruptures or holes. Cell disintegration was also observed due to the increase of membrane permeability (Figure 20). Chitosan BNMs also increased the reagent oxygen species (ROS) production in *F. oxysporum*, that can increase the oxidative stress, even cause the release of cytochrome C, leading to apoptosis (Figure 21) [84].

Chitosan–silver composite NPs were tested on *Danio rerio* and *Paratelphusa hydrodromous*. The predation of *D. rerio* larvae was not inhibited, it actually increased with approximately 19%. The superoxide dismutase (SOD) activity from contaminated hepatopancreas tissue of fresh water crab, *P. hydrodromous*, was stimulated. A possible explanation might be the enhancement of pre-existing enzyme or the synthesis of new enzymes [85]. 

There is a knowledge gap in the ecotoxicity of chitosan based BNMs, as available research is focused on applications, especially in medicine. There is data on the ecotoxicity of NMs capped with chitosan, but the data is also scarce. The effects of chitosan capped poly(*ε*-caprolactone) (CS-PCL) NPs were tested on *Daphnia similis*. No acute toxicity was observed for the CS-PCL NPs, but these could enhance the retention time of the NPs in the gut of the organism, prolonging the exposure to substances that would be loaded into these NPs, which could potentially be toxic [86]. 

Chitosan capped Ag NPs, at concentrations above 5 mg/L, induced cell division in the root meristem of *Allium cepa*. An increase in the total number of cells in prophase also occurred, which could be associated with a violation of the supramolecular structure of the chromosomes. Other observed effects were the presence of lagging chromosomes and the induction of polyploidy [87].

The effects of PBNMs of chitosan on plants include chromatin alterations and DNA damage, accumulation of hydrogen peroxide, synthesis of abscisic acid, increase in cytosolic Ca^2+^, oxidative burst, etc. [88]. In worms (*Tubifex tubifex*), a weight loss was recorded after treatment. The levels of metallothionein were significantly higher than in control. Glutathione, glutathione-S-transferase, glutathione-reductase and catalase activities were also increased for the chitosan PBNM treatment [89]

#### 4.1.2. Nanoalginic acid

**Figure 22:**
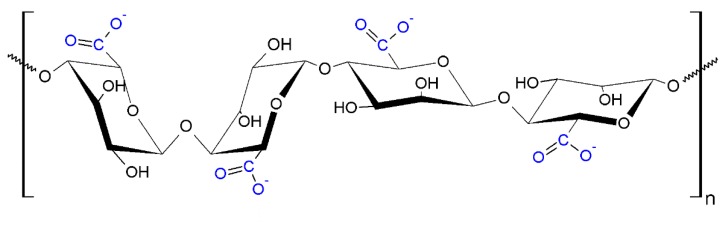
Structure of alginic acid pristine bionanomolecules.

Alginic acid is an anionic polysaccharide composed of two repeating units, forming a linear polymer: *D*-mannuronate and *L*-guluronate (Figure 22). It is a linear binary copolymer with homopolymeric blocks of either *D*-mannuronate, either *L*-guluronate, interspersed with alternating monomers. Due to its low toxicity, biocompatibility, biodegradability and mild gelation at addition of cation (ex. Ca^2+^), it is extensively investigated and used for many biomedical applications [77,90].

There is a lack of information regarding the ecotoxicity of both BNMs and PBNMs of alginate in the literature. The research topics mainly covered are the use of alginate in medicine [91,92,93,94], and its use in wastewater remediation [95,96,97,98].

There are articles that study alginate as PBNMs used in the reduction of ecotoxicity of other NMs or as dispersant materials. For example, alginate was studied as a dispersant in ecotoxicity of TiO_2_ NMs on embryos of zebrafish (*Danio rerio*). Alginate, with a z-average diameter of 178 nm, reduced the amount of TiO_2_ stuck to the glass vials, thus reducing the loss of the tested NMs, not by neutralizing the surface charge of nano-TiO_2_ (both TiO_2_ and alginate have negative charges), but through steric hindrance [99]. Alginate PBNMs were also tested for the reduction of ecotoxicity of TiO_2_ NMs on *Artemia franciscana* and *Phaeodactylum tricornutum*. The growth of *P. tricornutum* was not significantly affected by the presence of alginate in either the negative or positive control (the reference toxicant was (K_2_Cr_2_O_7_)) tests. The immobilization after 24 or 48 h of *A. franciscana* was not significantly affected by the presence of alginate in the negative control, but it made a significant difference in the positive control, where, at 24 h, alginate reduced the ecotoxicological effects of the reference toxicant (CuSO_4_ * 5H_2_O). Thus, alginate reduced the toxicity of CuSO_4_ * 5H_2_O, but not of K_2_Cr_2_O_7_, acting as a confounding factor. Alginate also reduced the bioavailability of TiO_2_ NMs in the *P. tricornutum* assay, a possible explanation being a capping or coating effect of the alginate. In the *A. franciscana* assay, no significant differences were observed between the TiO_2_ NMs with and without alginate [100].

One of the few data available on the ecotoxicity of alginate BNMs assesses the effects of chitosan–alginate nanoparticles on bullfrog (*Lithobates catesbeianus*) tadpoles, but not as its principal objective. The BNMs ecotoxicity is compared to clomazone pestanal® in its free form and associated with the chitosan–alginate nanoparticles. In all exposed groups (clomazone, BNMs and clomazone-BNMs) there was a significant increase of melanomacrophage centers, triggering a hepatic response in the tadpoles. Both groups exposed to BNMs presented hepatic sinusoids full of erythrocytes and abundant melanomacrophage centers which reflect that the BNMs might be recognized as toxin by the tadpole organism [101].

The ecotoxicity of alginate PBNM hydrogels was tested on two microalgae (*Halamphora coffeaeformis* and *Cylindrotheca closterium*) in correlation with the jellifying agent used: calcium chloride, copper sulfate or zinc acetate. All three metal ions caused an approximately 85% inhibition of algae adhesion, while the growth reduction was the highest for Cu^2+^, followed by Zn^2+^ [102].

The ecotoxicity of alginate PBNMS is indirectly assessed, not being the scope of the articles. For example, in the case of rainbow trout, the nitrogen digestibility and dry matter content of the feces were reduced, while the protein content, visceral and liver weights and the mortality were not influenced by alginate as PBNMs [103]. In cats, the intraperitoneal injection of alginate caused proteinuria and hematuria, the kidney tubules being occluded by erythrocytes. Both intravenous and intraperitoneal administration caused renal tubular damage, necrosis and fragmentation of the liver cells [104]. PBNMs of sodium alginate did not cause ecotoxic effects on *Ceriodaphnia cornuta*, as no mortality was observed in an indirect assessment [105].

#### 4.1.3. Nanocellulose

**Figure 23:**
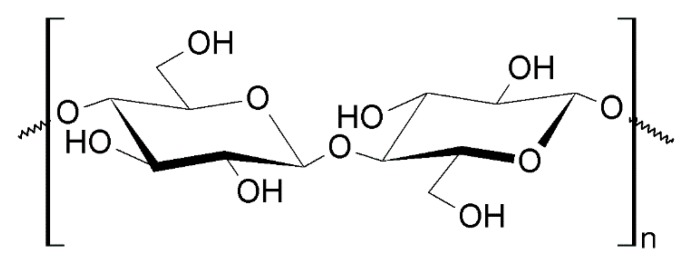
Structure of cellulose pristine bionanomolecules.

Cellulose is the most abundant natural polymer found in nature and it is mainly extracted from wood pulp [106,107]. It is a linear carbohydrate polymer, its monomeric units being *β*-*D*-glucopyranose molecules with *β* (1→4) covalent linkage (Figure 23). The length of the biopolymer chains present variations with the origin and treatment of the raw material. For example, in case of wood pulp, there are 300–1700 anhydroglucose units (AGUs), while plant fibers such as cotton and bacterial cellulose have 800–1000 AGUs. The numerous applications of cellulose and its derivatives, such as coatings, films, membranes, pharmaceuticals, etc., are due to its distinct fiber morphology. Elementary fibrils (1.5–3.5 nm lateral dimensions (LDs)), microfibrils (10–30 nm LDs) and microfibrillar bands (100 nm LDs) define the morphological hierarchy of cellulose [107].

Recently, considerable interest has been focused on finding new material applications for cellulose such as development of cellulose nanocrystals. Nanocrystalline cellulose can readily be obtained by subjecting native cellulose to strong acid hydrolysis, but the most common method of preparing cellulose nanomaterials is aqueous and solvent solution casting [106]. 

Due to their excellent mechanical properties, good biocompatibility and its renewable nature, cellulose BNMs are used in the fields of biomedical engineering and material science [108]. For example cellulose BNMs, such as cellulose nanocrystals, nanowhiskers [109] and nanofibers, are of interest for use in reinforcing fillers instead of cellulose fibers [106]. 

Since cellulose BNMs and PBNMs are biocompatible and biodegradable, their ecotoxicological effects are questionable. For example, cellulose BNMs caused no decrease of nematode (*C. elegans*) number [110]; caused mechanical inhibition of mobility in *D. magna* neonates [111]; had LC_50_ values higher than 1 g/L for *D. magna* and *O. mykiss*, and 0.3 g/L for *C. dubia* [112]; induced relatively low mortality or any other developmental impairment towards embryonic zebrafish [113]; etc. Cellulose in PBNM form showed no toxic effects toward *Pseudomonas putida*, *S. capricornutum*, *D. magna* and *D. rerio* [114]; showed toxicity to *Toxoptera graminum* [115]; etc.

#### 4.1.4. Nano polyhydroxyalkanoates (PHA)

**Figure 24:**
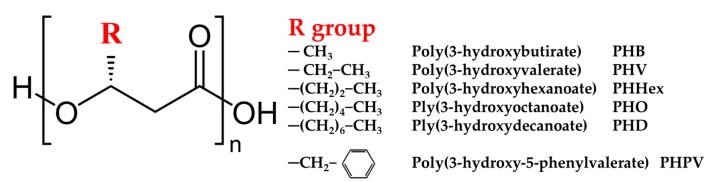
Structure of polyhydroxyalkanoate (PHA) pristine bionanomolecules.

Polyhydroxyalkanoates (PHA) are linear polyesters of hydroxyalkanoates (Figure 24), with a molecular weight in the range of 50–1000 kDa. All monomers units are in the *D*(-) configuration due to the biosynthetic enzymes that are stereospecific. These polymers are synthesized by gram negative and positive bacteria from at least 75 different genera, and are accumulated intracellularly, under conditions of nutrient stress, to levels as high as 90% of the cells’ dry weight, to act as carbon and energy reserve [116].

PHA are biocompatible, nontoxic and biodegradable, which promotes their applications as plastic replacement, in packaging, as chiral precursors for chemical synthesis of optically active compounds, as biodegradable carriers for medicine, drugs, herbicides and insecticides, as osteosynthetic materials for bone growth stimulations, etc. [116].

PHA nanomaterials are developed by bacterial direct synthesis, phase separation, as nanobiocomposites, etc., for different applications such as in medicine. For example, PHA nanofibrous matrices are developed for use as materials in cell growth supporting [117], or nanobeads of PHA produced for use as biomaterial in various applications in medicine [118]. PHA nanogranules have applications in fields such as drug delivery, bioseparation, enzyme immobilization, protein purification and vaccines [119].

There is a knowledge gap in the ecotoxicity of PHAs as both bionanomaterials and pristine bionanomolecules, highlighted by Hauser et al. in their environmental hazard assessment of polymeric and inorganic nanomaterials used in drug delivery [120]. The data available shows that PHA nanofibers are able to support the growth of rat neural stem cells [121]. Poly(3-hydroxybutirate) (P3HB) granules showed a decrease in concentration after dispersion in different organs in rats [122]. The degradation of PHAs in mammals happened gradually in six months or more, not causing weight loss in mice [123].

#### 4.1.5. Nano polylactic acid (PLA)

Polylactic acid (PLA) is an aliphatic polyester with its monomer being exclusively lactic acid. It is a biocompatible, bioresorbable and biodegradable polymer with high strength and thermoplastic properties [124,125]. Applications of PLA are as packaging materials [125], biomedical materials for implants, sutures, screws and plates, and in textile applications [124]. PLA is also used in tissue engineering as fixation device materials [126] and in drug delivery [127].

**Figure 25:**
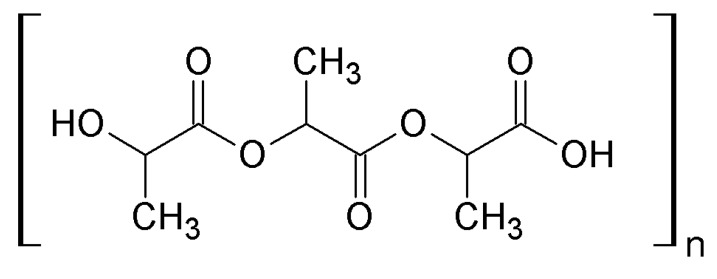
Structure of poly (*L*-lactic acid) (PLA) pristine bionanomolecules.

PLA can be produced from renewable resources, its monomer being mainly synthesized by bacteria from the genus *Lactobacillus*, through the Embden–Meyerhof pathway, predominantly from glucose (Figure 25). Some species synthesize the *L*(+)-isomer of lactic acid, such as *L. amylophilus* and *L. salivarius*, while other species, like *L. acidophilus* and *L. jensenii*, yield the *D* isomer [125]. The synthesis of PLA implies several steps, starting with the production of its monomer and ending with the polymerization of lactic acid. The polymerization can follow three main routes: 1. condensation polymerization; 2. azeotropic dehydrative condensation and 3. ring-opening polymerization [124].

Nanoparticles of PLA are used as delivery systems in medicine due to their low toxicity and hydrolytic degradability [124].

Hauser et al. highlighted the lack of information available on the ecotoxicity of bionanomolecules of polylactic acid [120]. The little available information reveals that PLA caused a slight increase in mitochondrial activity in mouse fibroblasts with a significant decrease of DNA synthesis [128], but it didn’t cause an acute or chronic inflammatory response in rats, with no polymer rejection by the organism [129].

### 4.2. Ecotoxicity of Carbon-Based Nanomaterials

Carbon-based NMs are mainly composed of carbon. The most common forms are hollow spheres and ellipsoids, referred to as fullerenes, or cylinders, known as carbon nanotubes (CNTs) [6].

#### 4.2.1. Carbon Nanotubes

The most studied carbon-based NMs are carbon nanotubes. These are built from sp^2^-hybridized carbon atoms assembled via very short *s*-bonds as aromatic rings. The rings are assembled according to a planar periodic lattice looking like a single-atom-thick hexagonal pavement [130].

CNTs have a wide range of properties, such as morphological (length, diameter, bundling) and structural (number of walls, metallic or semiconducting electrical behavior). CNTs, short or entangled, may be readily taken up by cells in a passive way, such as passive diffusion or by piercing the cell membrane, or in an active way through mechanisms like endocytosis or phagocytosis [130].

A production capacity that exceeds several thousand tons per year reflects a worldwide commercial interest in carbon nanotubes. The use of the CNTs extends over various application areas such as coatings and films (e.g., paints that reduce biofouling of ship hulls, thin-film heaters for the defrosting of windows), composite materials (e.g., electrically conductive fillers or flame-retardant additives in plastics), microelectronics (e.g., CNT thin-film transistors), energy storage (e.g., lithium ion batteries for notebook computers and mobile phones), environment (e.g., water purification) and biotechnology (e.g., biosensors, medical devices) [131].

The investigation of the potential impact of CNTs on the different environmental compartments (soil, water) is very important because CNT-containing materials may not be properly disposed in the absence of specific regulation. Initial studies on a single environment compartment revealed that biological species, such as crustaceans, worms, amphibian larvae, all interact with CNTs, which transit, without visible harm, through the gastrointestinal pathway. In most cases, at low concentration, less than 10 mg/L in aquatic studies, no significant effect is observed. The actual CNT concentrations in the environment are expected to be few orders of magnitude below this value of 10 mg/L, since only available data are based on calculations, upon hypotheses on the transfer between air, soil, and water. However, some toxicity is generally noticed at higher concentrations, which seems in most cases possible to correlate with ‘mechanical’ effects, such as perturbation of the digestion or the respiration related more to the presence of large amount of foreign material in the body [130].

The dispersion of CNTs can be directly modified by organisms. Protozoan cells, such as *Tetrahymena thermophila* and *Stylonychia mutilus*, that ingest multiwalled or single-walled carbon nanotubes (MWCNTs or SWCNTs), without any discrimination of bacterial food, excrete these as sedimented granules in micron size. Impaired ingestion of bacteria by phagocytosis (bacterivory) and impaired regulation of bacterial growth can occur both for parental cells and to the two daughter cells during cell division. Thus, the ingested CNT may affect protozoan food intake, and could be transferred between generations and move up the food chain [132].

In algae, the toxic effects of CNTs are due to direct contact with the surface. Thus, CNT shading and formation of algae–CNT agglomerates can inhibit algal growth, as studies on *Pseudokirchneriella subcapitata* and *Chlorella vulgaris* suggest [132].

The bioaccumulation of environmental contaminants, such as hydrophobic organic contaminants (HOC), can be influenced by the presence of CNTs. The bioaccumulation of HOC was significantly reduced in the presence of SWCNTs in *Streblospio benedicti*, a deposit/suspension feeding polychaete, while in the deposit-feeding meiobentic copepod *Amphiascus tenuiremis* the HOC bioaccumulation was less affected [132].

**Figure 26:**
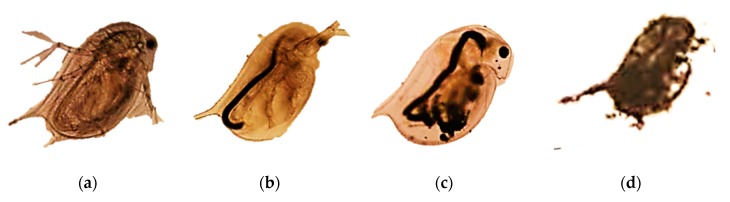
The uptake and adsorption of multi-wall carbon nanotubes (MWCNTs) by *Daphnia magna* after 48 h of exposure. (**a**) control; (**b**) 5 mg/L MWCNTs; (**c**) 50 mg/L MWCNTs and (**d**) 100 mg/L MWCNTs. (Modified after [75]).

The food processing of *Daphnia magna* was affected by the CNTs aggregated in the gut in the presence of food that contributed to the toxicity of the CNTs, which were, however, not able to cross the gut lumen. The presence of food reduced the elimination time of MWCNTs from a day to a few hours. The lipid coating of SWCNTs (that increase its water-solubility) was removed by the digestive system of *D. magna* making the CNTs less water soluble and more prone to sedimentation [132]. At high concentrations, MWCNTs adhered to the external surface of daphnids, being absorbed, and together with the ingestion of these materials, it was significant enough to cause sinking of daphnids to the bottom of the test vessels by the prevention of mobility through the water column. At low concentrations of MWCNTs, only ingestion was observed as shown in (Figure 26) [75].

The bioaccumulation of HOC or perfluorochemicals (PFC) in benthic larvae of *Chironomus plumosus* was reduced by the addition of MWCNTs [132].

In *Arabidopsis sp.* plants translation is affected by MWCNTs. The consequences are increased colonization of bacteria in infections, stress indication and root hair development inhibition [76].

The bioaccumulation of pyrene in terrestrial oligochaete *Eisenia foetida* was reduced by the presence of SWCNTs and MWCNTs in high concentrations, due to the increase of polycyclic aromatic hydrocarbons (PAH) elimination and of uptake by CNTs [132].

#### 4.2.2. Fullerenes

Fullerenes are a key topic nowadays in nanotechnology and industrial research due to their excellent unique properties, such as high symmetry. Fullerenes have a hexagonal ground state with sp^2^ bonding. The most symmetric molecule, with the largest number of symmetry operations is Buckminster C60 fullerene [6].

The use of the fullerenes extends over various application areas such as electronics (e.g., electrodes, solar cells) [133] or biology and medicine (e.g., antioxidants, antiviral agents, drug and gene delivery) [134]. The probability of environmental pollution with fullerenes increases due to the increased production and commercial applications. Thus, there is a considerable interest in the effects and behavior of carbon fullerenes in the environment [135].

The growth of the algae *P. subcapitata* was not inhibited by C60 fullerene, the EC_50_ being more than 90 mg/L, while fullerol even had a beneficial effect at 1mg/L, thus being a ROS scavenger [22].

C60 fullerene causes clubbing and tentacle retraction in chronic exposure in *Hydra attenuata* [11].

In mussels, such as *Mytilus edulis*, the accumulation of C60 fullerene took place in the digestive glands, followed by gills. At 0.1 and 1mg/L concentrations, several histopathological abnormalities were observed, such as necrosis in digestive tubules, hypoplasia in frontal and lateral cilia and atrophy in adductor muscle myocytes [22].

The heart rate of *D. magna* was increased by exposure to C60, along with the amplification of stereotypical movements in swimming and feeding and reduction of reproductive rates [11]. Acute exposure to C60 caused modulation of vertical position of daphnids over time and reduction of their swimming velocity, while chronic exposure caused cellular damage to the alimentary canal, delayed molting and inhibited reproduction [22].

In the nematode *Caenorhabditis elegans* hydroxylated fullerenes are able to induce apoptosis [136].

In higher organisms, such as fish and mammals, fullerenes can cross the membrane of eukaryote cells, as well as the blood–brain barrier, and can accumulate lysosomes and mitochondria [11].

#### 4.2.3. Graphene

Graphene is formed from strongly sp^2^-bonded carbon atoms that are arranged in a planar monolayer that forms a two-dimensional honeycomb lattice [137]. 

Graphene is used in various area such as field effects transistors, sensors, transparent conductive films, clean energy devices, etc. [138].

In *Euglena gracilis* graphene oxide (GO) induced growth inhibition, decrease of chlorophyll a content, but not of chlorophyll *b* and carotenoids. GO induced oxidative stress as the activities of catalase (CAT) and superoxide dismutase (SOD) were increased in comparison with control. There were no evident damages in the ultrastructure of the protozoa, however the cells were clearly covered with a layer of GO [139].

Fifty percent growth inhibition was induced by GO in Raphidocelis subcapitata, along with oxidative stress and membrane damage. GO also decreased the autofluorescence intensity, due to oxidative stress and/or a shading effect due to the agglomeration of GO [140].

**Figure 27:**
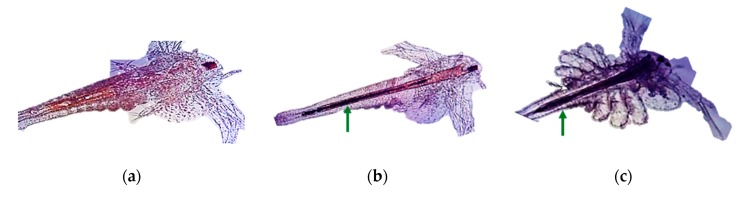
Light microscope images of (**a**) control, (**b**) pristine graphene monolayer flake (PGMF) and (**c**) graphene nanopowder grade C1 (GNC1-)treated *Artemia salina* (24 h exposure, 1.25 mg/L. Arrows indicate the presence of PGMF or GNC1 in the gut. (Modified after [141]).

No toxicity was observed by the exposure of *Artemia salina* to graphene at maximum concentrations, however the microscopical analysis showed the presence of pristine graphene monolayer flakes (PGMF) and graphene nanopowder grade C1 (GNC1) in the gut of the crustaceans (Figure 27). An altered pattern of oxidative stress biomarkers was observed at a 48-hour exposure. PGMF and GNC1 induced an increase in catalase and glutathione peroxidase activities, as well as an increase in the level of lipid peroxidation of membranes [141].

**Figure 28:**
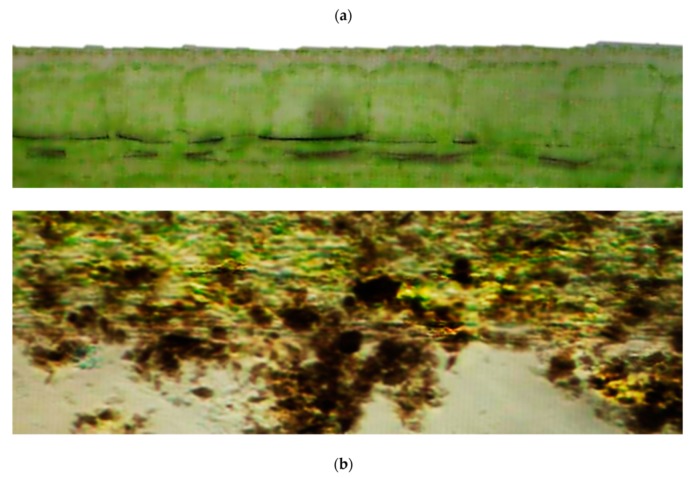
Roots of *Lemna minor* in (**a**) control and (**b**) treatment with 5 mg/L graphene oxide (GO). (Modified after [86]).

After a 96-hour co-exposure to Cu and GO, the roots of duckweeds were covered with GO fragments (Figure 28). GO significantly decreased the nutrient contents of *L. minor* only at concentrations of at least 5 mg/L [86].

### 4.3. Ecotoxicity of Metallic Nanomaterials

The exposure of plants to metallic nanomaterials causes nanotoxicity at physiological level such as root length inhibition, biomass decrease, altered transpiration rate, and plant developmental delays. The NMs that enter plant tissue cause the disruption of chlorophyll synthesis in leaves. Evidence of genotoxicity of metallic NMs to plants is provided by the analysis of the mitotic index, chromosomal aberrations, and micronuclei induction [62].

#### 4.3.1. Aluminum Nanomaterials

Aluminum based NMs have several industrial applications, such as absorbents, abrasives, desiccants etc., due to their excellent dielectric and abrasive properties. These highly adsorptive materials are, in contrast, chemically active and potentially hazardous to the environment [142].

It was observed that Al_2_O_3_ NMs decreased the viability of algal populations at short-term exposure, while at a long term exposure a gradual recovery was observed [15]. The exposure of aluminum NMs to *Scenedesmus obliquus* caused oxidative stress by altering the SOD activity and the concentrations of glutathione and malondialdehyde [76].

The toxicity of Al_2_O_3_ NMs to *D. magna* is dose-dependent and it is higher than its bulk material’s. The potential ecotoxicity and environmental health effect of these NMs cannot be neglected as these are ingested by the daphnids [75].

The toxicity of aluminum NMs to plants is species dependent as these had no obvious effect on cucumber, significantly retarded root elongation of ryegrass and lettuce and promoted the root growth of radish and rape [143].

Ni/*γ*-Al_2_O_3_ (NiNC) nanoceramics were analyzed by the AMPHITOX assay on the larvae of the *Rhinella arenarum* toad. The sublethal effects were mainly hyperkinesia and reduced swimming movements, an expression of behavioral alteration, as well as collapsed cavities, edema and axial flexure. The contents of Al and Ni were higher in heads than in tails, and these elements were found in the oral disc [142].

#### 4.3.2. Cerium Nanomaterials

Cerium oxide nanomaterials (CeO_2_ NMs) have a wide range of applications in engineering and biomedical manufacturing industries and have the ability to act as a redox catalyst, thus it may be able to both induce or alleviate oxidative stress in organisms [144].

The potential toxicity of ceria NMs was assessed on the unicellular alga, *Chlamydomonas reinhardtii*, by unbiased transcriptomics and metabolomics approaches to provide insight into molecular toxicity pathways. Although the ceria NMs were internalized in *C. reinhardtii* into intracellular vesicles, no significant toxicity was observed on the algal growth at any concentrations. The only effects, such as downregulation of photosynthesis and carbon fixation with associated effects on energy metabolism, were observed at ceria NM-concentrations higher than environmental levels [145].

**Figure 29:**
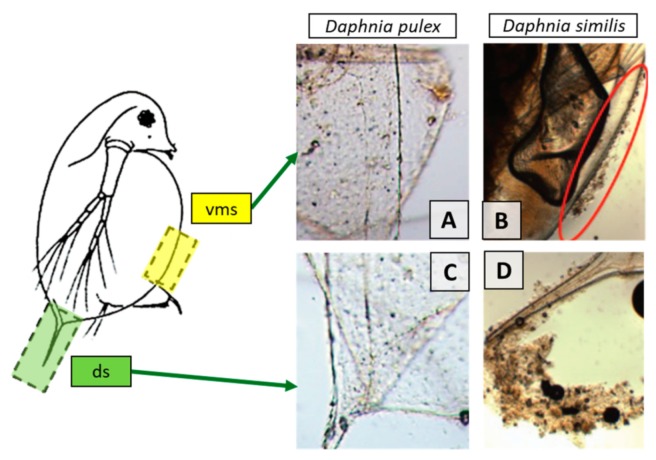
Representative image of distal spine (ds) and ventral margin of the shield (vms) in *Daphnia pulex* and *D. simillis* exposed to 10 mg/L of CeO2 NPs for 48 h. Note the accumulation of particles onto the cuticle of *D. simillis*. (Modified after [146]).

*Daphnia similis* and *Daphnia pulex* were used for the assessment of toxicity of CeO_2_ NMs. *D. similis* was 350 times more sensitive to the ceria NMs than *D. pulex* in acute ecotoxicity assessment. The NMs were absorbed on both species, but less strongly on *D. pulex*, and for both species the swimming velocities (SV) were differently and significantly affected. The different toxicity for the two species can be explained by the differences in morphology, such as the presence of reliefs on the cuticle and a longer distal spine in *D. similis* acting as traps for the CeO_2_ aggregates (Figure 29). *D. similis* also has double swimming velocity than *D. pulex*, thus it can collide with twice as much NMs [146].

**Figure 30:**
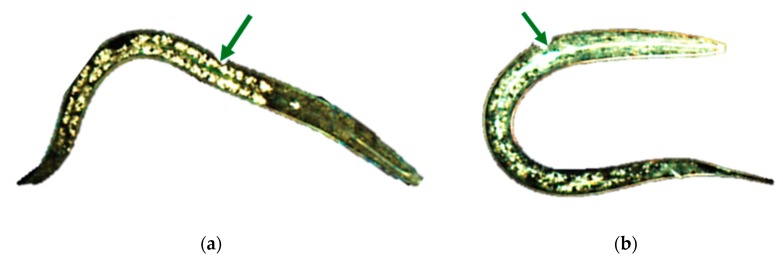
Representative dark field image of (**a**) control wild-type nematode (green arrow indicates the light scattered by tissue aggregates from nematode extraintestinal tissues) and of (**b**) a wild-type nematode exposed to 12.5 mg/L CeO2 NPs for 24 h (green arrow indicates ingested CeO2 NPs). (Modified after [144]).

The toxic effect on the nematode *C. elegans* was dose-dependent growth inhibition. Mildly altered growth was observed for some metal and oxidative stress-sensitive mutant nematode strains in comparison with the wild-type. *C. elegans* ingested CeO_2_ NMs but these materials were not detected inside the nematode cells. The aggregation of NMs around bacterial food and/or inside the gut tract may cause, at least in part, the growth inhibition, due to the inhibition of feeding caused by these aggregates (Figure 30) [144].

#### 4.3.3. Cadmium Nanomaterials

Cadmium based quantum dots (QDs), also called colloidal semiconductor nanocrystals, have several uses in biomedical applications, such as fluorescent biosensors (used in the detection of proteins, nucleic acids, etc.) and in bio-imaging (cellular or in vivo targeting and imaging) [147].

**Figure 31:**
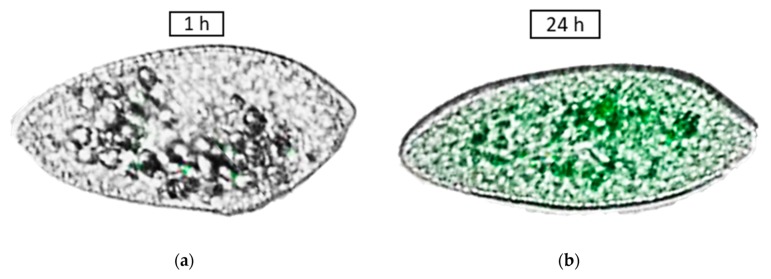

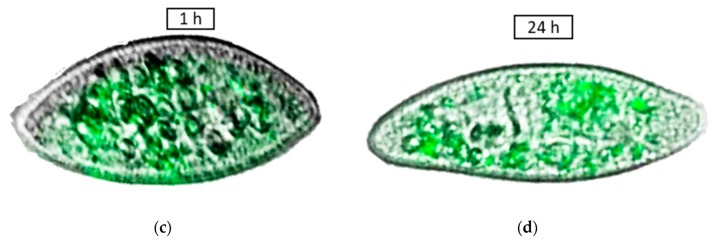
Fluorescence-based measurement of accumulation of cadmium telluride quantum dots (CT QDs) in *Paramecium* cells. Superimposed (fluorescence and bright field) images of (**a**) and (**b**) control *Paramecium* (exposed with *E. coli*) at (**a**) 1 and (**b**) 24 h; and (**c**) and (**d**) of treated *Paramecium* (*E. coli* + QDs) at (**c**) 1 h and (**d**) 24 h. (Modified after [148]).

The effects of cadmium telluride quantum dots (CdTe QDs) were assessed on a microbial food chain, composed of Escherichia coli as prey and *Paramecium caudatum* as predator. It was observed that the QDs caused the loss of the bacterivory potential of paramecium, including an ∼12 h delay in doubling time. When paramecium was exposed to the QDs (25 mg/L at 24 h), these NMs were bioaccumulated, as shown by the fluorescence based stoichiometric analysis (Figure 31) [148].

**Figure 32:**
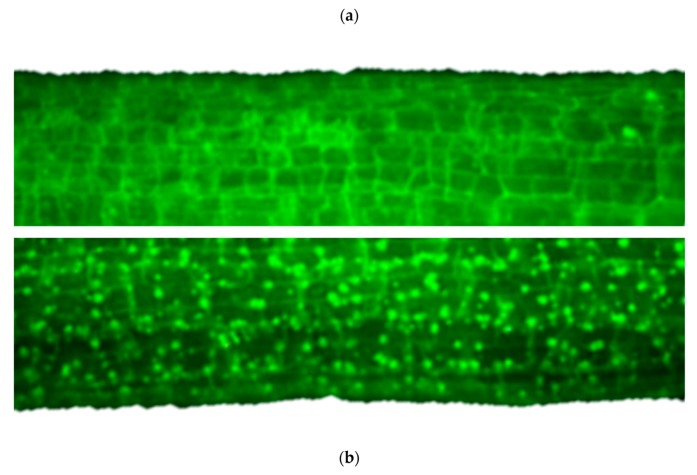
Fluorescence microscopic images of roots of (**a**) control and (**b**) treated *Spirodela polyrhiza* plant after four days of exposure to *L*-Cysteine-capped CdS NPs.(B) indicates the remarkable presence of nanoparticle aggregates inside the root tissues (modified after [149]).

*L*-Cysteine-capped CdS NMs expressed a high uptake in the roots of *Spirodela polyrhiza*. This was confirmed by epifluorescence microscopy where the presence of NMs was observed inside the root tissues (as particles with different sizes in intracellular spaces), the NMs aggregates appearing as optically dense signals under fluorescence (Figure 32). The entrance of NMs into roots is done through intercellular plasmodesmata, capillary forces, osmotic pressure, pores in cell walls, or via the highly regulated symplastic route [149].

Cadmium telluride quantum dots (CdTe QDs) caused abnormal foraging behavior (related to the altered function of the motor neurons) in *C. elegans*, at long-term early onset exposure. Thus, a decrease in fluorescence of the motor neurons cell bodies was observed, indicating an alteration in their development [136]. The main route of exposure of nematodes to QDs was determined, by fluorescence microscopy, to be through the digestive tracts [150].

#### 4.3.4. Cobalt Nanomaterials

Cobalt NMs are of great interest in both life-sciences and industry, due to their wide range of applications, such as in lithium-ion batteries, gas sensors or medicine [151].

Decline in the growth rate and reduction in biomass concentration of two cyanobacteria, *Microcystis* and *Oscillatoria*, was observed at exposure to Co NMs. Other observed effects were the reduction of carotenoid, protein and carbohydrates contents and the decrease of SOD activity with increase of NMs concentration in the microalgae [152].

*Allium cepa* was used to investigate the effects of cobalt oxide NMs as an indicator organism. The observed phytotoxic effect at root level was the inhibition of root elongation due to the massive adsorption of NMs into the root system [153].

#### 4.3.5. Copper Nanomaterials

Due to their potential applications in diverse fields, such as biomedicine, electronics, and optics, copper NMs have been the focus of intensive study [154].

When present in high concentrations, copper is supposed to be highly toxic in aquatic systems, causing irreversible damage [9]. Due to their antimicrobial and biocidal properties, copper oxide (CuO) NMs are frequently used. These NMs may represent an important source of contamination in the aquatic environment, due to their application in antifouling paints used on boats and immersed structures [155].

The dissolution and adsorption of CuO NMs onto cell walls, of the prokaryotic alga *Microcystis aeruginosa,* was observed to be enhanced by dissolved organic carbon (DOC). The cell walls were crossed by the NMs through the cell wall pores and the cell plasma membrane was crossed via endocytosis, thus the NMs reaching the thylakoids and granules [22]. In *L. minor*, the CuO NMs alter the activity of antioxidative enzymes such as guaiacol peroxidase, glutathione reductase and ascorbate peroxidase, increasing necrosis and bleaching [76].

A mesocosm that modeled tidal cycles was used for the assessment of CuO NMs toxicity on the worm *Hediste diversicolor* and the clam *Scrobicularia plana*. In both organisms, the observed effects were oxidative stress defense system responses (affected oxidative stress markers were: glutathione *S*-transferase (GST) and catalase (CAT)) and induction of genotoxicity (comet assay was used to asses DNA damage) [15].

The effects of Cu NMs were assessed on cowpea, *Vigna unguiculata*; specifically, how NMs affect the ascorbate peroxidase (APX), catalase (CAT), superoxide dismutase (SOD), glutathione reductase (GR) activities, lipid peroxidation, Cu uptake and bioaccumulation in roots, leaves and seeds [76].

#### 4.3.6. Gold Nanomaterials

Due to their functions in medicine and therapeutics, in electronics, catalysis, cosmetic, and food industries, gold NMs are of special interest [156].

The exposure of marine bivalves to Au NMs was assessed. In *Scrobicularia plana*, Au NMs formed aggregates and gold was accumulated in the soft tissues of the clams. Biochemical effects of NMs were metallothionein induction, increase in catalase, superoxide dismutase and glutathione S-transferase activities (indicating defense against oxidative stress), while a behavioral effect was the impairment of the burrowing behavior [156]. In *Mytilus edulis*, Au NMs enhanced stress parameters in digestive glands, mantle and hematocytes, paradoxically protecting from the oxidative stress due to menadione [11].

The vegetative uptake of gold NMs was assessed using as a model poplar plants *Populus deltoides × nigra.* The Au NMs were observed in the cytoplasm and various organelles of root and leaf cells, and these accumulated in the plasmodesma of the phloem complex in root cells suggesting that the transport between cells and translocation throughout the whole plant were done with ease, inferring the potential for entry and transfer in food webs [157].

#### 4.3.7. Iron Nanomaterials

Iron NMs have a wide range of applications such as magnetic, electrical, catalytic and biomedical (e.g., MRI contrast enhancer) [158]. These NMs are also suitable for immobilization and degradation of soil contaminants due to their high specific surface area and high reactivity. Thus, their use in soil clean-up purposes could cause potential hazards to soil organisms and macrophytes [64].

Severe negative effects of nanosized zero valent iron (nZVI) were observed on *Heterocypris incongruens*, an ostracod, and on *Folsomia candida*, a collembolan. The effects were observed after seven days and prolonged exposure led to the oxidation of nZVI, reducing its toxicity [75].

A test battery, composed from algae (*Pseudokirchneriella subcapitata, Chlamydomonas sp.*), crustaceans (Daphnia magna), plants (*Raphanus sativus, Lolium multiflorum*) and worms (*Eisenia fetida, Lumbriculus variegatus*), was used for the assessment of the effects of nZVI. The testing of the iron NMs was difficult due to their turbidity, aggregation and sedimentation behavior in aqueous media, but nZVI proved to be toxic. The observed effects for plants were the inhibition of root elongation in *Raphanus sativus* and *Lolium multiflorum* [97]. 

**Figure 33:**
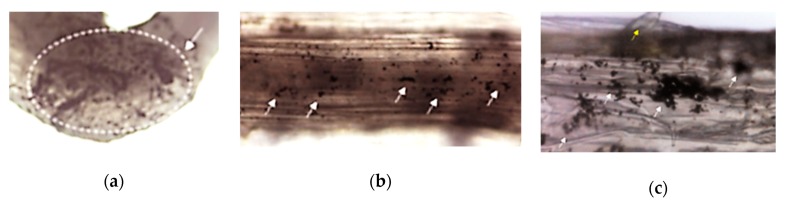
Bright-field micrographs illustrating (**a**) root apex of *Lepidium sativum* and (**b**) and (**c**) root longitudinal sections of (**b**) *Sinapis alba* and (**c**) *Sorghum saccharatum* treated with 992 mg/L of *n*Fe for 72 h at 25 °C. White arrows indicate *n*Fe aggregates, while yellow one shows root hairs. (modified after [64]).

The effects of iron-based NMs were tested on three plant species: *Lepidium sativum*, *Sinapis alba* and *Sorghum saccharatum*. Microscopy images show that the NMs aggregated on the surface of the plants, visible as black spots, sometimes even forming a coating. The accumulation of iron NMs inside the tissue was observed in longitudinal sections of roots (Figure 33). However, the NMs did not enter the palisade cells or the xylem, as shown in transverse sections [64]. 

#### 4.3.8. Platinum Nanomaterials

Platinum NMs have a wide range of applications in fields such as CO oxidation, hydrogen or methanol fuel cells, electrochemical oxidation of ethanol or formic acid, oxygen reduction and glucose detection [159].

Antioxidant effect of Pt NMs coated with polyvinylpyrrolidone was observed in larvae of *C. elegans* (L4 development stage). The observed effects were counteraction of induction of oxidative stress by paraquat (an intracellular free radical-generating compound) and prolongation of life span of wild-type and short-living mutant mev-1 worms [11]. The life span of wild-type N2 nematodes was also extended, regardless of thermotolerance or dietary restriction. The NMs reduced the accumulation of lipofuscin (an endogenous autofluorescent marker that increases in concentration with oxidative stress) and ROS induced by paraquat. The effects of Pt NMs were compared to EUK-8, a superoxide dismutase (SOD)/catalase mimetic. The results showed similar results for the two tested substances, suggesting that Pt NMs are a superoxide dismutase (SOD)/catalase mimetic [160].

#### 4.3.9. Silver Nanomaterials

The major applications of Ag NMs include their use as optical sensors, catalysts, in engineering, optics, electronics, and, most importantly in the biomedical field, as a bactericidal and therapeutic agent [161].

The effects of Ag NPs were tested on two microalgal species: *Dunaliella tertiolecta* and *Chlorella vulgaris*. The observed effects were depletion of chlorophyll content, inhibition of photosystem II (PSII) electron transport, membrane damage via lipid peroxidation possibly via ROS-mediated processes [22].

In adult *Mytilus edulis*, poly(allyl)amine (PAAm)-capped silver NPs caused the formation of a donut shaped microstructures on the nacreous layer of the bivalve, which can be explained by the disturbance of the shell calcification process. In the oyster *Crassostrea virginica*, Ag NPs caused the inhibition of embryo development and the destabilization of the lysosomal membrane of hepatopancreas cells of adults [22].

In *D. magna*, silver NPs accumulated in the gut, under the carapace, in the brood chamber and on the antennae and body surface, affecting their swimming behavior [11,22].

The effects of silver NMs on the nematode *C. elegans* were neurotoxicity, reduction of velocity, flex, amplitude, and wavelength of the body bend of exposed worms and reduction of survival and reproductivity [136].

#### 4.3.10. Titanium Nanomaterials

Titanium dioxide NMs are frequently used in the production of paper, plastics, paints, cosmetics and welding rod coating material [162].

The effects of TiO_2_ NMs on the algae *P. subcapitata* were light shading, interference on nutrient uptake through adsorption onto the cell surfaces and production of ROS which cause lipid peroxidation of cell membrane, leading to leaching of DNA from the algal cells. In *Chlamydomonas reinhardtii*, TiO_2_ NMs caused up-regulation of genes associated with antioxidant activities, such as superoxide dismutase (SOD), glutathione peroxidase (GPX), catalase (CAT) and plastid terminal oxidase (ptox2), but these did not alter the transcriptions of genes associated with photosynthesis and carotenoid biosynthesis [22].

In *D. magna* TiO_2_ NMs caused the reduction of brood size and body length, disruption of digestive enzymes, such as amylase and esterase, affecting nutrient assimilation and energy allocation. The increase of GST, GPX, and CAT activities demonstrate the induction of oxidative stress by titanium NMs [22].

In *C. elegans*, it was found that proteins involved in oxidative stress protection and metal elimination, such as SOD isoforms, metallothioneins and heat shock proteins, have a key role in resistance to TiO_2_ NPs. These NPs led to a substantial decrease in both head thrash or body bend in nematode mutants (SOD-2, SOD-3, metallothionein-2 and heat shock protein-16.48) compared to the wild type [136].

The symbiosis of *Rhizobium leguminosarum* bv. *viciae* 3841 on garden peas *Pisum sativum* was affected by TiO_2_ NMs by delaying root nodule formation and the onset of nitrogen fixation [76].

#### 4.3.11. Zinc Nanomaterials

Zinc oxide NMs have been extensively used in products like coatings, paints and sunscreens due to their chemical stability and strong adsorption ability. These also have a high inherent risk of water contamination, being able to reach high concentrations in surface waters posing significant threat to aquatic ecosystems [163].

**Figure 34:**
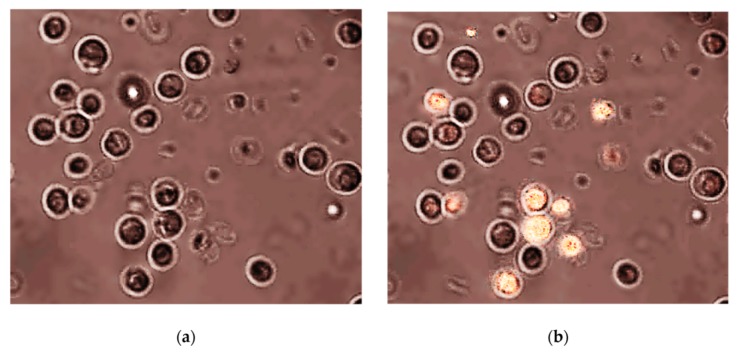
Confocal images of (**a**) untreated cell with intact membrane, preventing entry of PI (propidium iodide) dye, resulting in unstained cells and (**b**) cells treated with 300 mg/L ZnO NPs (zinc oxide nanoparticles) for 72 h showing PI stained cells due to compromised membrane integrity. (Modified after [163]).

The ZnO NMs at lower concentration cause toxic effects, such as decrease of cell viability (Figure 34) due to compromised membrane integrity, on algae mainly due to the Zn ions. However, at higher concentrations, the growth of algae less sensitive to Zn ions, such as *Phaeodactylum tricornutum*, was inhibited, as the contact between algal cells and NM particles increased [22,163].

The feeding and defecating rates of the snail *Lymnaea stagnalis* were suppressed by ZnO NMs, 86% of the Zn being retained inside the organism. The reproduction and survival of copepods was also altered by ZnO NMs, along with the impairment of their movement [15,22].

**Figure 35:**
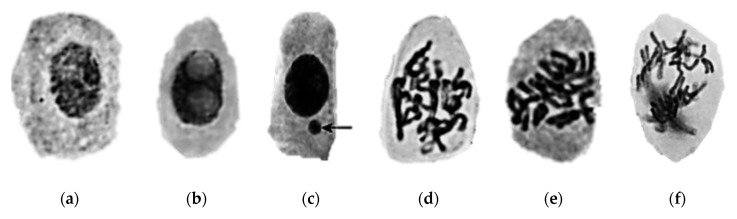
Chromosomal aberrations observed in *Allium cepa* meristematic cells exposed to ZnO NPs. (**a**) Normal cell in prophase; (**b**) binucleated cells at early telophase; (**c**) prophase nucleus with micronucleus; (**d**) disturbed metaphase; (**e**) disturbed anaphase and (**f**) multipolar anaphase. (Modified after [143]).

Genotoxicity and cytotoxicity were the effects of exposure of *Allium cepa* to ZnO NPs. The mitotic index was inhibited in a concentration dependent way, indicating a mitodepressive effect of the NPs, which may prevent several cells from entering the prophase and blocking the mitotic cycle during interphase inhibiting DNA/protein synthesis (Figure 35). The presence of ZnO NP deposits inside the cell matrix of *A. cepa* is shown in microscopy images confirming their internalization and agglomeration [143]. 

### 4.4. Comparison of Ecotoxicity of Described NMs Based on Their Half Maximal Effective Concentration Values

In order to compare the ecotoxicity of NMs and to classify these NMs into toxicity classes, from published data, the half maximal effective concentrations (EC_50_) were identified and analyzed. The concentration values were taken into account only for the ecotoxicity tests selected as being adaptable for nanomaterials. In order to be able to compare the concentrations of different types of nanomaterials, one model species was selected for each test, because the concentrations show interspecific variations. The selected model species were:

- Aquatic tests
  ○ algae assay—*Raphidocelis subcapitata*
  ○ duckweed assay—*Lemna minor*
  ○ daphnid assay—*Daphnia magna*
- Terrestrial tests
  ○ plant assay—*Allium cepa*
  ○ nematode assay—Caenorhabditis elegans
  ○ earthworm assay—*Eisenia foetida*

In the ecotoxicity comparison tables (Table 6 and Table 9), for both the aquatic and terrestrial environments, only nanomaterials that had EC_50_ values for at least one of the tests are presented, even if these represent one of the mentioned special cases. The low number of nanomaterials described in the comparison tables is due to the fact that some, although they are studied from an ecotoxicological point of view, either do not present the results in the form of EC_50_, or they are realized on species other than those chosen as a model.

Where EC_50_ values were presented for multiple NM types based on the same substance, which might differ in shape and/or size, the mean value of all types of tested NMs was included in the comparison table. The average EC_50_ values were calculated as a mean of the concentration of each aquatic and terrestrial assay in order to compare the NMs, but only for those NMs that had available data for all assays, for the rest of NMs “not applicable” (N/A) was entered in the table. The data that was not an exact value but was represented as greater or smaller than a value, the value +1 and –1, respectively, was taken into calculation.

#### 4.4.1. Comparison of Ecotoxicity of NMs in the Aquatic Environment

The aquatic ecotoxicity of substances during short term exposure (acute toxicity), for all organisms types (including algae, plants, invertebrates and fish) is divided into five toxicity categories (Table 5), according to both U.S. Environmental Protection Agency [164] and United Nations [165].

Only cadmium, copper, iron, silver and titanium-based NMs had available data regarding their EC_50_ values for all the selected assays and model test species. By comparing the average EC_50_ values for all assays, silver NMs are the most toxic (VHT/A1.1), followed by the highly toxic (HT/A1.2) copper NMs, the moderately toxic (MT/A2) cadmium-based NMs and the slightly toxic (ST/A3) iron and titanium NMs. The order of their toxicity is: Ag NMs > Cu NMs > Cd NMs > Ti NMs > Fe NMs.

The most sensitive aquatic assay for NMs is the algae assay (according to data for *Raphidocelis subcapitata*), while the least sensitive is the duckweed assay (according to data for *Lemna minor*) as shown by the average EC_50_ values per assay.

The EC_50_ values for aquatic ecotoxicity assays suitable for NMs of some nanomaterials were classified into the five toxicity categories for the aquatic environment (Table 7).

For algae, the most toxic NMs were those based on silver, gold and iron, and the least toxic were the carbon nanotubes and graphene. The most toxic NMs to duckweed were silver and platinum NMs, iron NMs being the least toxic. For daphnids, the highest toxicity was observed for silver NMs, followed by cadmium and platinum NMs, the least toxic being cerium NMs and carbon nanotubes.

#### 4.4.2. Comparison of Ecotoxicity of NMs in the Terrestrial Environment

The terrestrial ecotoxicity of substances during short term exposure (acute toxicity), for both plants and soil dwelling invertebrates, is divided into three toxicity categories (Table 8), according to [177].

The EC_50_ values for terrestrial ecotoxicity assays suitable for NMs of some nanomaterials were compared (Table 9).

Only silver NMs had available data regarding their EC_50_ values on all the selected assays and model test species. The silver NMs were the most toxic towards nematodes, followed by plants and earthworms. By considering the earthworm assay, where all NMs that were taken into account had EC_50_ values, the order of toxicity for the NMs is: Ag NMs > Zn NMs > Cu NMs > Ce NMs.

The most sensitive terrestrial assay, based on the scarce available data, was the nematode assay (according to data for *Caenorhabditis elegans*), followed by the plant assay (according to data for *Allium cepa*) and the earthworm assay (according to data for *Eisenia foetida*).

The EC_50_ values for terrestrial ecotoxicity assays suitable for NMs of some nanomaterials were classified into the three toxicity categories for the terrestrial environment (Table 10).

For nematodes the most toxic NM was based on silver, and the least toxic on titanium. For earthworms, the most toxic NM was based on silver as well, and the least toxic on cerium.

By comparing EC_50_ values of silver nanomaterials obtained in aquatic and terrestrial ecotoxicity tests, it can be observed that aquatic tests are more sensitive than terrestrial ones.

## 5. Final Remarks

Bibliometric investigations revealed that the research on ecotoxicity, nanomaterials and the ecotoxicity of nanomaterials increased during the period 2010–2019. This highlights the need for further research into the ecotoxicity of nanomaterials.

The ecotoxicological effects of a potentially polluting substances such as nanomaterials can be analyzed by applying ecotoxicity tests. Such tests have been developed over time, creating standard test guides.

Although these standards use a wide range of organisms, from the simplest, such as algae and invertebrates, to the most complex, such as higher plants and vertebrates, only the use of simple organisms is recommended in the first phase, and of the most complex only in case of need.

This trend has been applied by us as well, only SSRET (suitable for simple and rapid ecotoxicity testing) organisms being considered and included in our study regarding the testing of nanomaterial ecotoxicity.

Due to the special properties of nanomaterials with respect to their bulk material and their wide range of applications, it is essential to test the ecotoxicity of NMs. The use of standard tests for testing the ecotoxicity of nanomaterials is possible but requires some adaptation. We considered as adaptive tests only those that use only SSRET organisms, are suitable for nanomaterials (i.e., were applied and adapted to NMs in at least ten scientific articles), have a shorter duration than 30 days, do not require special equipment or training and have low costs. Also, the possibility of these tests being high throughput is an advantage.

The tests considered to be adaptable for nanomaterials are the algal, duckweed, amphipod, daphnid and chironomid tests in the aquatic environment, and the terrestrial plants, nematodes and earthworm tests in the terrestrial environment. 

The adaptations of these tests to nanomaterials include the methods of preparing the material solutions, as well as different methods of preparing the culture media, quantifying the number of organisms, etc.

**Figure 36:**
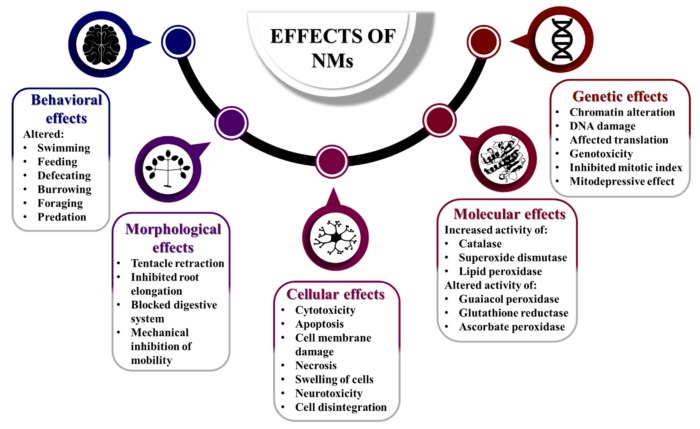
Summary of effects of different categories that NMs can have on different types of organism. The described effects can occur in all or only a few organism types.

Analyzing the different effects of nanomaterials on SSRET and other organisms, it can be observed that these effects are complex and are of different categories (Figure 36). NMs can physically affect organisms, for example by blocking the digestive tract or by attaching to the surface of the body. Also, they may have effects at the cellular level, by breaking the plasma membrane, at the molecular level, by inducing the production of different enzymes, or at the genetic level, by altering the chromosomes. The behavior of some organisms might also be affected, by inducing abnormal feeding or swimming behaviors.

Comparing the EC_50_ values of some nanomaterials obtained in aquatic and terrestrial ecotoxicity tests, that were selected as conforming for nanomaterials and are conducted on selected model species, it can be observed that such values are available mainly for aquatic ecotoxicity. It can also be observed that from the three selected aquatic tests, the algae, duckweed and daphnids tests, the most sensitive was the algae test, while the least sensitive was the duckweed test. From the selected terrestrial tests, the plant, nematode and earthworm tests, the most sensitive was the nematode test, the least sensitive being the earthworm test.

It can also be observed that in aquatic tests only for nanomaterials based on cadmium, copper, iron, silver and titanium EC_50_ values were described for all three selected tests. The toxicity order of these four NMs was Ag > Cu > Cd > Ti > Fe. The most toxic NMs to algae were silver, gold and iron, to duckweed and daphnids silver, while the least toxic to algae were graphene and carbon NTs, to duckweed iron, and to daphnids carbon NT.

In the terrestrial tests only silver NMs had EC_50_ values for all the selected assays. Comparing the concentration for the earthworm assay, the toxicity order of NMs was possible: Ag > Zn > Cu > Ce. 

In conclusion, the assessment of the ecotoxicity of nanomaterials and its mechanism are essential, as well as the adaptation of the standard ecotoxicity testing methods for nanomaterials. There are still difficulties regarding the testing of NMs, but these could be resolved by further research on this subject. It is clear that the effects of nanomaterials on different types of organisms are complex, thus these must be further analyzed.

## Figures and Tables

**Table 1:** Summary of bibliometric analysis of research on ecotoxicity of nanomaterials (NMs) from Web of Science Core Collection (Web of Science, Clarivate Analytics) presented in this review.

Ecotoxicity	Nanomaterials	Ecotoxicity of NMs	Ecotoxicity of NMs by Type
1. Ecotoxicity by topic (Figure 1a)	4. Nanomaterials by topic (Figure 1b)	6. NMs vs. ecotoxicity of NMs by topic (Figure 1c and Figure 15)	7. Ecotoxicity of types of NMs by topic (Figure 17)
2. Ecotoxicity by title vs. by topic (Figure 2)	5. Types of NMs by topic (Figure 5)		8. Categories of NMs and ecotoxicity of NMs by topic (Figure 16)
3. Aquatic vs. terrestrial ecotoxicity by topic (Figure 3)			

**Table 2:** Algae, plants and invertebrates used in standard ecotoxicity assays.

LE ^1^	OT ^2^	Organism Category	Common Name of Group	Species	Standard Guidelines
Aquatic	Plants	Algae	Cyanobacteria	*Anabaena flos-aquae* *Synechococcus leopoliensis* *Microcystis aeruginosa*	OECD 201; EPA 850.4500; 850.4550; ISO 8692; 10253; 11044; ASTM E1218-04; GC EPS1/RM/25;
			Diatoms	*Navicula pelliculosa* *Skeletonema costatum* *Thalassiosira pseudonana* *Phaeodactylum tricornutum*	
			Green algae	*Raphidocelis subcapitata (Pseudokirchneriella subcapitata; Selenastrum capricornutum)* *Desmodesmus subspicatus (Scenedesmus subspicatus)* *Dunaliella tertiolecta*	
			Red algae	*Ceramium tenuicorne*	ISO 10710;
		Angiosperms	Duckweed	*Lemna minor* *Lemna gibba* *Spirodela polyrhiza*	OECD 221; EPA 850.4400; ISO 20079; 20227; ASTM E1415-91; GC EPS1/RM/37;
			Great Manna grass	*Glyceria maxima*	OECD 239;
			Watermilfoil	*Myriophyllum spicatum* *Myriophyllum aquaticum*	OECD 238; 239; ISO 16191;
	Animals	Snails	Mud snail	*Potamopyrgus antipodarum*	OECD 242;
			Pond snail	*Lymnaea stagnalis*	OECD 243;
		Bivalves	Clams	*Mercenaria mercenaria*	EPA 850.1025; 850.1055; 850.1710; ISO 17244; ASTM E2455-06; E724-98;
			Mussel	*Mytilus edulis* *Mytilus galloprovincialis*	
			Oyster	*Crassostrea virginica* *Crassostrea gigas*	
		Oligochaetes	Blackworms	*Lumbriculus variegatus*	OECD 225; 315;
			Tubificid worms	*Tubifex tubifex* *Branchiura sowerbyi*	
		Crustaceans	Amphipods	*Gammarus fasciatus* *Gammarus pseudolimnaeus* *Gammarus lacustris* *Ampelisca abdita* *Eohaustorius estuaries* *Rhepoxynius abronius* *Leptocheirus plumulosus* *Hyalella azteca*	EPA 850.1020; 850.1735; 850.1740; ISO 16712; GC EPS1/RM/26; EPS1/RM/33; EPS1/RM/35;
			Copepods	*Acartia tonsa* *Nitocra spinipes*	ISO 14669; 16778; 18220; ASTM E2317-04;
			Mysids	*Americamysis bahia (Mysidopsis bahia)*	EPA 850.1035; ASTM E1191-03a; E1463-92;
			Ostracods	*Heterocypris incongruens*	ISO 14371;
			Penaeids	*Farfantepenaeus aztecus (Penaeus aztecus)* *Farfantepenaeus duorarum (Penaeus duorarum)* *Litopenaeus setiferus (Penaeus setiferus)*	EPA 850.1045;
			Water fleas	*Daphnia magna* *Daphnia pulex* *Ceriodaphnia dubia*	OECD 202; 211; EPA 850.1010; 850.1300; ISO 6341; 10706; 20665; ASTM E1193-97; E1295-01; GC EPS1/RM/11; EPS1/RM/14; EPS1/RM/21;
		Insects	Chironomids	*Chironomus riparius* *Chironomus yohimatsui* *Chironomus tenans* *Chironomus dilutus*	OECD 218; 219; 233; 235; EPA 850.1735; GC EPS1/RM/32;
Terrestrial	Plants	Angiosperms	Monocots	*Allium cepa* *Avena sativa* *Hordeum vulgare* *Lolium perenne* *Oryza sativa* *Secale cereal* *Sorghum bicolor* *Triticum aestivum* *Zea mays*	OECD 208; 227; EPA 850.4100; 850.4150; 850.4230; 850.4300; 850.4800; ISO 11269-1; 11269-2; 17126; 18763; 21479; 22030; ASTM E1963-09; GC EPS1/RM/45; EPS1/RM/56;
			Dicots	*Beta vulgaris* *Brassica campestris var. chinensis* *Brassica napus* *Brassica oleracea var. capitate* *Brassica rapa* *Cucumis sativus* *Daucus carota* *Fagopyrum esculentum* *Glycine max (Glycine. soja)* *Gossypium sp.* *Helianthus annuus* *Lactuca sativa* *Lepidium sativum* *Linum usitatissimum* *Lotus corniculatus* *Phaseolus aureus* *Phaseolus vulgaris* *Pisum sativum* *Raphanus sativus* *Sinapis alba* *Solanum lycopersicum (Lycopersicon esculentum)* *Trifolium pratense* *Trigonella foenum-graecum* *Vicia sativa*	
	Animals	Snails	Helicidae	*Helix aspersa aspersa*	ISO 15952;
		Nematodes		*Caenorhabditis elegans*	ISO 10872; ASTM E2172-01;
		Oligochaetes	Earthworms	*Eisenia foetida* *Eisenia andrei*	OECD 207; 222; 317; EPA 850.3100; ISO 11268-1; 11268-2; 11268-3; 17512-1; 23611-1; ASTM E1676-12; GC EPS1/RM/43;
			Potworms	*Enchytraeus buchholzi* *Enchytraeus albidus* *Enchytraeus crypticus* *Enchytraeus luxuriosus*	OECD 220; 222; 317; ISO 16387; 23611-3; ASTM E1676-12;
		Mites		*Hypoaspis aculeifer (Geolaelaps aculeifer)*	OECD 226;
		Springtails		*Folsomia candida* *Folsomia fimetaria*	OECD 232; ISO 11267; 17512-2; GC EPS1/RM/47;
		Insects	Beetles	*Oxythyrea funesta*	ISO 20963;
			Bumble bees	*Bombus sp.*	OECD 246; 247;
			Honeybees	*Apis mellifera*	OECD 213; 214; 237; 245; EPA 850.3020; 850.3030; 850.3040;
			Leaf cutter bees	*Megachile rotundata*	EPA 850.3040;
			Sweat bees	*Nomia melanderi*	EPA 850.3040;
			Flies	*Musca autumnalis* *Scathophaga stercoraria*	OECD 228;

^1^ LE = life environment; ^2^ OT = organism type

**Table 3:** Vertebrates used in standard ecotoxicity assays.

Organism Type	Life Environment	Common Name	Standard Guidelines
Vertebrates	Aquatic	fish	OECD 203; 204; 210; 212; 215; 229; 230; 234; 236; 240; 305; EPA 850.1075; 850.1400; 850.1730; ISO 10229; 22082; 7346-1; 7346-2; 7346-3; 12890; 15088; 23893-1; 23893-2; 23893-3; ASTM E1192-97; E729-96; E1241-05; E1711-12; GC EPS1/RM/09; EPS1/RM/10; EPS1/RM/13; EPS1/RM/22; EPS1/RM/28;
		amphibians	OECD 231; 241; ISO 21427-1; ASTM E1192-97; E2591-07; E729-96;
	Terrestrial	birds	OECD 205; 206; 223; EPA 850.2100; 850.2200; 850.2300; ASTM E857-05;
		mammals	EPA 850.2400; ASTM E1163-10; E1619-11;

**Table 4:** Analysis of ecotoxicity assays that correspond to the criteria described above. For simplification of table only tests less than 30 days are showed.

LE ^1^	OT ^2^	Organism	Test	NMAP ^3^	SAT ^4^	TD ^5^
Aquatic	Plants	Algae	Growth inhibition	✓	✗	72 h
			Toxicity	✓	✗	96 h
		Duckweed	Growth inhibition	✓	✗	7 days
			Toxicity	✓	✗	
		Watermilfoil	Sediment free toxicity	✗	✗	14 days
			Water-sediment toxicity	✗	✗	
	Animals	Mud snails	Reproduction	✗	✗	28 days
		Pond snails	Reproduction	✗	✗	28 days
		Bivalves	Acute toxicity	✗	✗	48–96 h
		Oligochaetes	Sediment-water toxicity	✗	✗	28 days
		Amphipods	Acute toxicity	✓	✗	96 h
			Spiked whole sediment toxicity	✓	✗	10 days
		Mysids	Acute toxicity	✗	✗	96 h
		Penaeids	Acute toxicity	✗	✗	96 h
		Water fleas	Acute immobilization	✓	✗	48 h
			Acute toxicity	✓	✗	48 h
			Chronic toxicity	✓	✗	21 days
			Reproduction	✓	✗	21 days
		Chironomids	Acute immobilization	✓	✗	48 h
			Sediment-water toxicity with spiked sediment	✓	✗	20–28 days
			Sediment-water toxicity with spiked water	✓	✗	20–28 days
			Spiked whole sediment toxicity	✓	✗	10 days
Terrestrial	Plants	Angiosperms	Early seedling growth toxicity	✓	✗	14 days
			Seedling emergence/Seedling growth	✓	✗	14–21 days
			Vegetative vigor	✓	✗	21–28 days
	Animals	Nematodes	Toxicity	✓	✗	96 h
		Earthworms	Acute toxicity (contact and soil)	✓	✗	14 days
			Subchronic toxicity	✓	✗	28 days
		Mites	Reproduction	✗	✗	14 days
		Springtails	Reproduction	✗	✗	21–28 days
		Bumblebees	Acute contact toxicity	✗	✗	48–96 h
			Acute oral toxicity	✗	✗	48–96 h
		Honeybees	Acute contact toxicity	✗	✗	48–96 h
			Acute oral toxicity	✗	✗	48–96 h
			Chronic oral toxicity	✗	✗	10 days
			Larval toxicity	✗	✗	7 days
			Toxicity of residue on foliage	✗	✗	24 h
		Flies	Developmental toxicity	✗	✗	18–23 days

^1^ LE = life environment; ^2^ OT = organism type; ^3^ NMAP = nanomaterial adaptation possibility; ^4^ SAT = special apparatus and training; ^5^ TD = test duration.

**Table 5:** Toxicity categories for aquatic ecotoxicity.

Categories According to U.S. EPA [164]	Categories According to U.N. [165]	EC_50_ (mg/L)
Very highly toxic (VHT)	Acute 1.1 (A1.1)	< 0.1
Highly toxic (HT)	Acute 1.2 (A1.2)	0.1–1
Moderately toxic (MT)	Acute 2 (A2)	> 1–10
Slightly toxic (ST)	Acute 3 (A3)	> 10–100
Practically nontoxic (PNT)	Acute 4 (A4)	> 100

The EC_50_ values for aquatic ecotoxicity assays suitable for NMs of some nanomaterials were compared (Table 6).

**Table 6:** Comparison of EC_50_ values for some NMs and classification in aquatic toxicity categories. The classification into toxicity categories is color-coded as follows: values that are in the VHT/A1.1 category have dark red shading, in HT/A1.2 have red shading, in MT/A2 have orange shading, in ST/A3 have yellow shading and in PNT/A4 have green shading.

NMs Based on:	EC_50_ Value (mg/L) for Aquatic Organisms			Tox. Cat. for Mean EC_50_ for All Assays
	Algae 72 h Test	Duckweed 168 h (7 d) Test	Daphnid 48 h Test	
Carbon NT	29.9 [166]	NDA	>100 [23]	N/A
Fullerene	NDA	NDA	11 [167]	N/A
Graphene	20 [140]	NDA	20 [167]	N/A
Cerium	NDA	NDA	52.42 ** [168]	N/A
Cadmium	3.5 * [169]	0,45 [170]	0.33 * [169]	1.427
Copper	0.7 [23]	0,84 [171]	0.9 [23]	0.813
Gold	0.048 [172]	NDA	>30 [23]	N/A
Iron	0.07 [173]	>100 [173]	43.41 [173]	48.16
Platinum	NDA	0.213 [174]	0.444 [174]	N/A
Silver	0.003 [23]	0,03 [175]	0.003 [23]	0.012
Titanium	6.8 [23]	>90 [176]	29.5 [176]	42.433
Zinc	0.14 [23]	NDA	1.87 [23]	N/A
AVERAGE EC_50_	6.795	32.255	24.323	

NDA=no data available; N/A not applicable; * if there was no data on EC_50_, the value of other concentrations, such as IC_50_ or median lethal concentration (LC_50_), were entered in the table; ** if there was no data regarding EC_50_ at the standard test time interval, the value of the concentrations at other time intervals were entered in the table;

**Table 7:** Classification of NMs into the five aquatic toxicity categories based on their EC_50_ values.

Toxicity Category	EC_50_ (mg/L)	Algae Assay	Duckweed Assay	Daphnid Assay
Very highly toxic	< 0.1	Ag, Au, Fe NMs	Ag NMs	Ag NMs
Highly toxic	0.1–1	Zn, Cu NMs	Pt, Cd, Cu NMs	Cd, Pt, Cu NMs
Moderately toxic	> 1–10	Cd, Ti NMs	-	Zn NMs
Slightly toxic	> 10–100	Graphene, Carbon NT	Ti NMs	Fullerene, graphene, Ti, Au, Fe, Ce NMs
Practically nontoxic	> 100	-	Fe NMs	Carbon NT

**Table 8:** Toxicity categories for terrestrial ecotoxicity.

Toxicity Categories	EC_50_ (mg/kg Soil Dry Weight)
Very toxic (VT)/Acute 1 (A1)	≤ 10
Toxic (T)/Acute 2 (A2)	> 10 – ≤ 100
Harmful (H)/Acute 3 (A3)	> 100 – ≤ 1000

**Table 9:** Comparison of EC_50_ values for some NMs and classification in terrestrial toxicity categories. The classification into toxicity categories is color-coded as follows: values that are in the VT/A1 category have red shading, in T/A2 have orange shading and in H/A3 have yellow shading.

NMs Based on:	EC_50_ Value (mg/kg Soil Dry Weight) for Terrestrial Organisms			Tox. Cat. for Mean EC_50_ for All Assays
	Plants 72 h Test	Nematodes 24 h Test	Earthworms 28 d Test	
Cerium	NDA	NDA	294.6 [178]	N/A
Copper	NDA	NDA	197 [179]	N/A
Silver	12.973 [180]	2.553 [181]	31 ** [182]	15.508
Titanium	NDA	18 [183]	NDA	N/A
Zinc	NDA	NDA	179 *** [184]	N/A

NDA=no data available; N/A not applicable; * if there was no data on EC_50_, the value of other concentrations, such as IC_50_ or LC_50_, were entered in the table; ** if there was no data regarding EC_50_ at the standard test time interval, the value of the concentrations at other time intervals were entered in the table; *** if there was no data regarding EC_50_ for the selected model species, but there were values for species of the same genus, these values were entered in the table.

**Table 10:** Classification of NMs into the three terrestrial toxicity categories based on their EC_50_ values.

Toxicity Categories.	EC_50_ (mg/kg Soil Dw)	Plant Assay	Nematode Assay	Earthworm Assay
Very toxic	≤ 10	-	Ag NMs	-
Toxic	> 10 – ≤ 100	Ag NMs	Ti NMs	Ag NMs
Harmful	> 100 – ≤ 1000	-	-	Zn, Cu, Ce NMs
